# Untargeted Metabolomic Approach to Determine the Regulatory Pathways on Salicylic Acid-Mediated Stress Response in *Aphanamixis polystachya* Seedlings

**DOI:** 10.3390/molecules27092966

**Published:** 2022-05-06

**Authors:** Kanakarajan Vijayakumari Rakhesh, Sunkarankutty Nair Ashalatha, Karthikeyan Mahima, Venkidasamy Baskar, Muthu Thiruvengadam

**Affiliations:** 1 Department of Botany, University of Kerala, Thiruvananthapuram 695581, India; rakhesh.kv@gmail.com (K.V.R.); ashabot2010@gmail.com (S.N.A.); 2 Department of Pharmacognosy, Siddha Central Research Institute, Chennai 600106, India; mahimakarthikeyan863@gmail.com; 3 Department of Biotechnology, Sri Shakthi Institute of Engineering and Technology, Coimbatore 641062, India; baskarbt07@gmail.com; 4 Department of Crop Science, College of Sanghuh Life Science, Konkuk University, Seoul 05029, Korea

**Keywords:** abiotic stress, Aphanamixis, metaboanalyst, metabolite expression, salicylic acid, terpenoid

## Abstract

Plants thrive under abiotic and biotic stress conditions with the changes in phytohormones like salicylic acid (SA), resulting in the synthesis of secondary metabolites. The present study determines the response of plants in producing secondary metabolites towards different SA concentrations at varying time intervals. Liquid chromatography-mass spectrometry-based metabolomics studies in *Aphanamixis polystachya* (Wall.) Parker seedlings are grown at 10 mM, 50 mM, and 100 mM SA concentrations, showed the differential expression of metabolites towards the stress. Alkaloids like amaranthin showed a 15-fold increase on the second day, and analog of androvinblastin showed a 20-fold increase on the sixth day in 10 mM SA compared with other groups. Flavanoid cyanidin 3-3 glucosyl was found to be with a 22-fold increment along with terpenoids betavulgaroside (18-fold), asiaticoside (17-fold), mubenin B (20-fold), and deslanoside (22-fold) increment in 50 mM SA on the sixth day. The shock exerted by 100 mM was too harsh, and the lowered metabolite production level was insufficient for the seedlings to survive at this higher SA condition. Arrangement of stressed groups using Pearson correlation studies, principal component analysis, and partial least square analysis placed 10 mM SA and controlled group closer and 50 mM SA and 100 mM SA groups closer to each other. The study observed that SA regulates metabolites that mediate biotic stress responses at low concentrations, and higher concentrations regulate abiotic stress regulating metabolites.

## 1. Introduction

Plants undergo various kinds of environmental stress due to their sessile nature. During stress, plants maintain the internal homeotic balance through fluctuation in pathways responsible for metabolite production [1]. These metabolites are the final products of genes that diversify shape, texture, signaling molecules, reserve food materials, color, aroma, etc. Proper identification of the mechanisms behind these gene expressions will provide the functional genomics of the plants. To the extent, proteomics and transcriptomics helped identify the underlining mechanisms in certain model organisms [2,3]. These studies only provide a snapshot of expressed genes for the given time and offer a predicted result. The higher number of mRNA sequences in transcriptome data would not promise its expression to protein. Similarly, the enzymatic activity of a translated protein depends on sheer chance. In this scenario, metabolomic profiling provides the most available information in all the omics strategies [4].

Like other omics studies, metabolomic studies were pioneered in *Arabidopsis* plants. The metabolic changes in the mutant and wild-type *Arabidopsis* plants were determined using Gas chromatography-Mass Spectrometry (GC-MS) based untargeted approach [5]. In this method, the data obtained is processed with various software, annotated, and the statistical techniques relative expression of metabolites and functional profile were easily obtainable [6]. Apart from this, metabolic snapshots of plants followed by correlation analysis will provide the basis for constructing metabolic networks. These statistical parameters deliver valuable insights into the altered behavior of various metabolites [7]. In the medicinal plant *Populus*, wild and mutant plants were grouped, and these strategies identified significant metabolites. PCA (Principal component analysis) and OPLS (Orthogonal partial least square) analysis with S-plot became useful in determining medicinally important metabolite production changes in wild and mutants [8].

Besides mutant study, metabolic changes in biotic and abiotic stress conditions on various plants were analyzed using Liquid chromatography–Mass spectroscopy (LC-MS) techniques. Chemical change during mycelia production by *Fusarium* species on plants during infection was identified by the Metlin database (Scripps Research Institute) search of LC-MS data [9]. In wheat cultivars, metabolites activated during drought regulation were determined using untargeted metabolomic studies. Differential expressions of fats, sugars, amino acids, and intermediate metabolites were identified and adopted to determine the pathways expressed in different cultivars [10]. Similarly, waxes were exclusively selected and quantified in wheat cultivars to identify drought-resistant phenotypes [11].

During abiotic or biotic stress, the plant evades the adverse condition successfully through the action of phytohormones like Salicylic acid (SA). SA was one of the essential phytohormones activated during the stressed condition, and it was known to maintain internal homeostasis. SA played a crucial role in plant development, disease resistance, and stress tolerance [12,13]. SA acted as an endogenous signaling mediator during the stress condition and was higher on different plants. SA compensates for the stress effect by itself or in association with other phytohormones such as jasmonic acid, abscisic acid, ethylene, gibberellins, auxins, etc. [14]. Several investigations indicated that SA influenced gas exchange parameters and water composition [15], increased phenolics accumulation [16], enhanced oxidative stress tolerance [17] and may alleviate osmotic stress [18]. In biotic stress, reactive oxygen intermediates (ROIs) have an essential role in defending pathogens along with SA. The ROIs were produced in the plasma membrane of affected cells and converted to H_2_O_2_. H_2_O_2_, together with SA, diffused into cells and induced the defense response in plants [19].

The biotic and abiotic stress to the seedling stages tends to be more destructive since fewer metabolites and pathways are expressed than mature ones. Plants evade these by increasing the chance of using a higher number of seed sets and a lower life cycle. While this might be helpful to herbs and shrubby plants, the survival of trees becomes affected due to a higher life span and lower seed sets. Seedlings of trees survive with the activation of pathways responsible for secondary metabolites in mature plants, and the studies on *Eucalyptus* seedlings can observe this. In heat-stressed *Eucalyptus* seedlings, altered metabolite expressions help them survive the adverse condition [20]. Since SA increased the metabolite content, studies on its effect on stress response in trees were absent. Hence, the present study aims to determine the effect of SA in maintaining the stress response in the tree species. A tree with well-characterized metabolites and a higher seed mortality rate will help determine SA during the stress response. Thus, *A. polystachya* was selected as the candidate for the study to fulfill the aim. *Aphanamixis polystachya* is an evergreen forest tree that belongs to the Meliaceae family and has a high percentage of seed mortality (>95%). *Aphanamixis* contains many secondary metabolites in which terpenoids are predominant, followed by flavonoids and alkaloids [21]. Many compounds like aphanamixin [22], aphapolin A and B [23], aphagranol [24], polystanin, aphanalide, nemordisin, meliasenin, and agladupols [25], aphanamene C [26], rohithukine [27], amooranin [28], and aphanin [29] were isolated and characterized from this tree. To determine the action of SA in stressed conditions, *A. polystachya* seedlings were grown in a controlled condition and induced with SA. The exogenous induction of SA will mimic the stress response effect in seedlings, and metabolomics profiling was carried out to determine its impact.

Similarly, the aim could be obtained by filling the objective of characterizing the metabolic changes during stress response in tree species upon the exogenous administration of SA. To determine the action of SA in stressed conditions, *A. polystachya* seedlings were grown in a control condition and induced with SA. The exogenous induction of SA will mimic the stress response effect in seedlings, and metabolomics profiling was carried out to determine its impact. Previous studies of several plant species justified the rationale behind the administration of exogenous SA in the present study. SA action on increment in the production of metabolites was observed in the hairy root culture of *Brugmansia candida*, with increased production of alkaloids [30]. In *Arabidopsis*, SA at lower concentrations mimics the gene expression patterns of pathogen attack. With the progression of time, the gene expression and concentration of secondary metabolites increased [31]. SA-induced stress in *Salvia miltiorrhiza* cell culture showed an increase in phenolic compounds [32]. Studies using exogenous SA administration showed higher stress responses, and SA-treated seedlings showed increased vigor and survival rate [33].

## 2. Results

### 2.1. Effect of Stress on Seedlings

**Figure 1:**
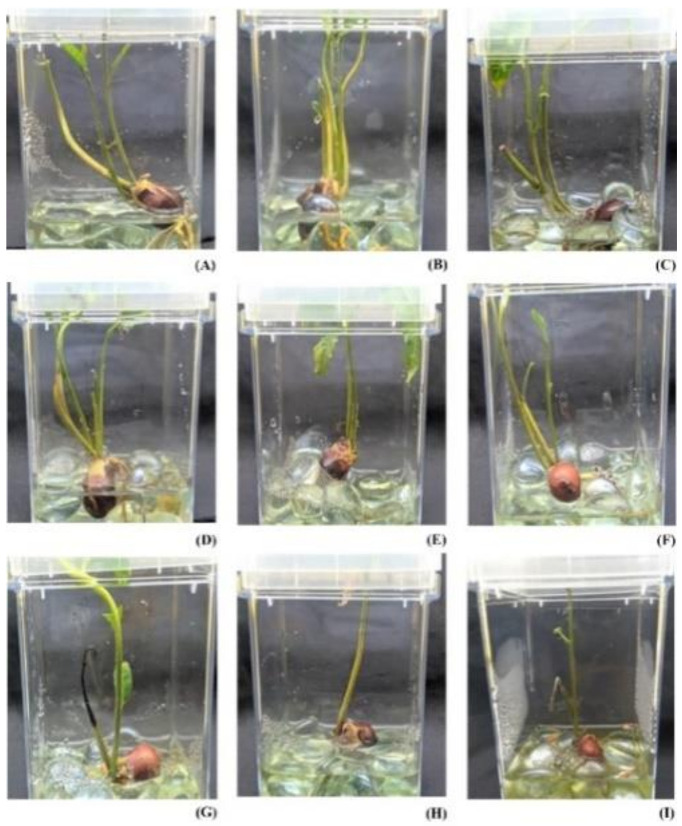
Experiment setup for Salicylic acid (SA) stress induction in *A. polystachya* seedlings (**A**) Seedling in 10 mM SA stress at Day 2. (**B**) Seedling in 10 mM SA stress at Day 4. (**C**) Seedling in 10 mM SA stress at Day 6. (**D**) seedling in 50 mM SA stress at Day 2. (**E**) Seedling in 50 mM SA stress at Day 4. (**F**) Seedling in 50 mM SA stress at Day 6. (**G**) Seedling in 100 mM SA stress at Day 2. (**H**) Seedling in 100 mM SA stress at Day 4. (**I**) Seedling in 100 mM SA stress at Day 6.

The response to stress was different in seedlings at 10 mM (milli Molar), 50 mM, and 100 mM concentrations of SA (Figure 1). Seedlings that were grown in 10 mM SA on the 2nd day (treated_1), 4th day (treated_2), and 6th day (treated_3) showed minimal changes in their appearance. On the other hand, seedlings at 50 mM SA on the 2nd day (treated_4), 4th day (treated_5), and 6th day (treated_6) showed yellowing of leaves as the days progressed. Seedlings in 100 mM on the 2nd day (treated_7), 4th day (treated_8), and 6th day (treated_9) showed severe stress symptoms with necrosis and leaf falls during the progression of time.

### 2.2. Determination of Metabolite Fraction in the Stressed Condition

**Figure 2:**
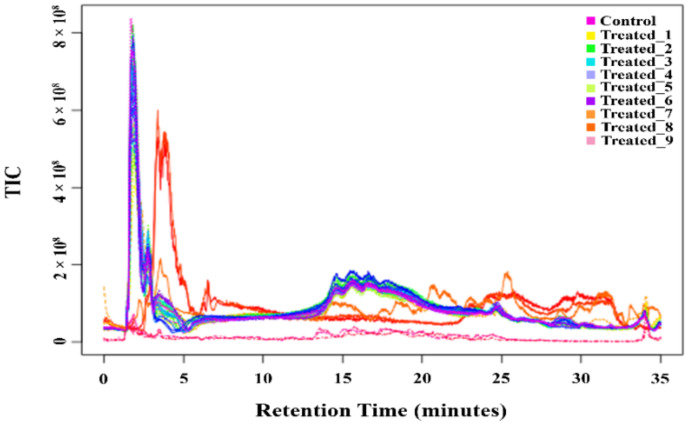
LC-MS Total ion chromatogram of *A. polystachya* seedling under control and all SA treatment conditions.

**Figure 3:**
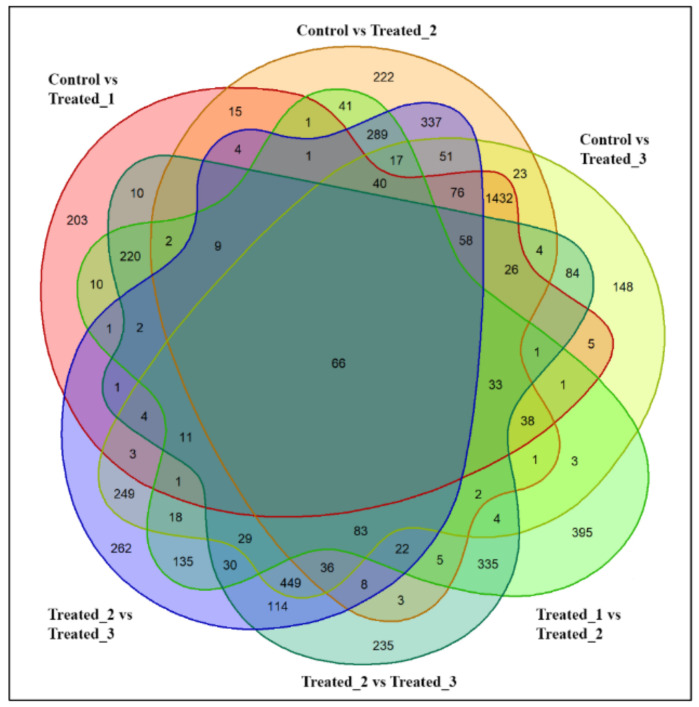
Venn diagram showing comparative expression of metabolites during 10 mM SA stressed condition in *A. polystachya* seedling.

The total ion chromatogram of the LC-MS study is represented in Figure 2. A total of 4298 metabolites were detected in all the experimental conditions. In 10 mM stressed condition, 2274 metabolites were expressed on treated_1 compared with the control. On treated_2, the number of metabolites revealed increased to 2945, and on the treated_3, it was around 2996. With the comparison of the stressed groups and control, an increment in the metabolite number was visualized. The Venn diagram (Figure 3) shows the metabolites’ distribution scenario during all the stress conditions. In control vs. treated_1, about 203 metabolites were unique for that group. In control vs. treated_2 showed 222, and the control vs. treated_3 showed 148 novel metabolites. Upon comparing the stressed group with each other, Treated_1 vs. Treated_2 showed 395 unique metabolites. The number of uniquely expressed metabolites increased from 235 in Treated_1 vs. Treated_3 to 262 in Treated_2 vs. Treated_3. In all the groups, a total of 66 metabolites were shared.

**Figure 4:**
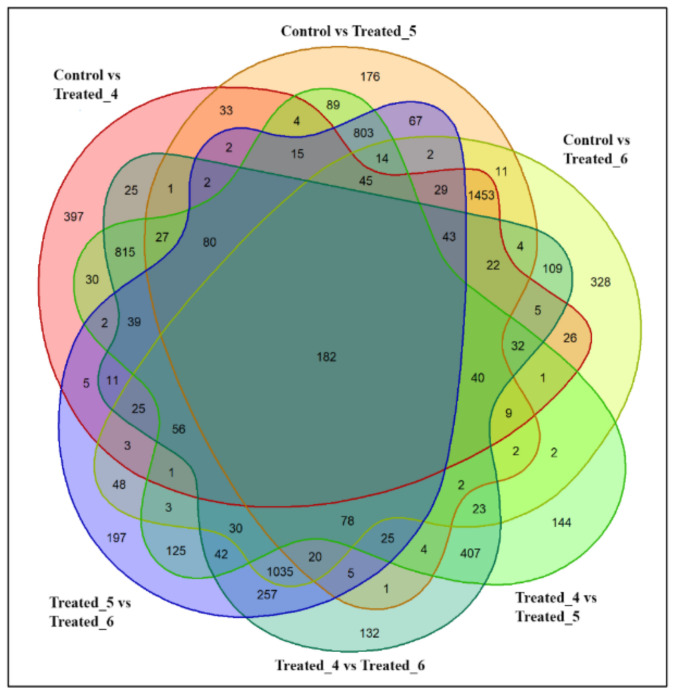
Venn diagram showing comparative expression of metabolites during 50 mM SA stressed condition in *A. polystachya* seedling.

In the 50 mM stressed condition, comparison with control and treated_4 showed the expression of 3406 metabolites. Control and treated_5 showed the expression of 3290 metabolites, whereas control and treated_6 showed around 3683 metabolites. Comparing the metabolites distribution in all the groups (Figure 4) showed 182 metabolites common in all experimental classes. Some metabolites are expressed uniquely in each group, and the Venn diagram depicts these metabolite numbers. In control and treated_4, around 397 metabolites were expressed, whereas control vs. treated_5 and treated_6 showed 176 and 328 unique metabolites, respectively. In experimental groups, treated_4 and treated _5 showed 144 metabolites, whereas treated_5 and treated_6 showed 197. A comparison between treated_4 and treated_6 revealed around 182 unique metabolites.

**Figure 5:**
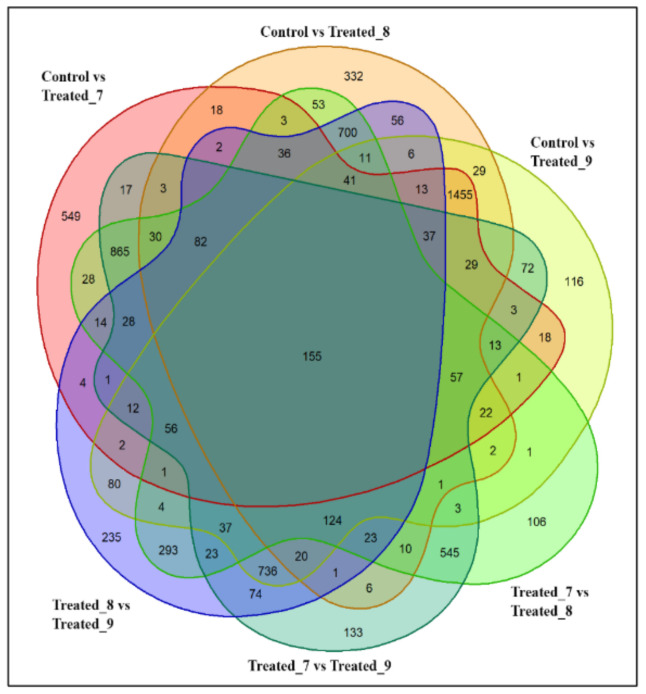
Venn diagram showing comparative expression of metabolites during 100 mM SA stressed condition in *A. polystachya* seedling.

In 100 mM stressed condition, in comparison with control, treated_7 showed an expression of 3595 metabolites, whereas treated_8 and treated_9 showed a lesser number, which is around 3357 and 3157, respectively. Comparing metabolites in all the experimental groups showed about 155 common metabolites (Figure 5). Uniquely expressed metabolites in each experiment class and overlapping class were projected in the Venn diagram. Control vs. treated_7, treated_8, and treated_9 showed 549, 332, and 116 uniquely expressed metabolites. The unique metabolites revealed that between treated_7 and treated_8 there were about 106, whereas treated_8 and treated_9 showed 235 metabolites. In the case of treated_7 and treated_9, there were approximately 133 unique metabolites expressions for the group.

### 2.3. Differential Production of Metabolites during the Stressed Condition and Identification of Significant Metabolites

**Figure 6:**
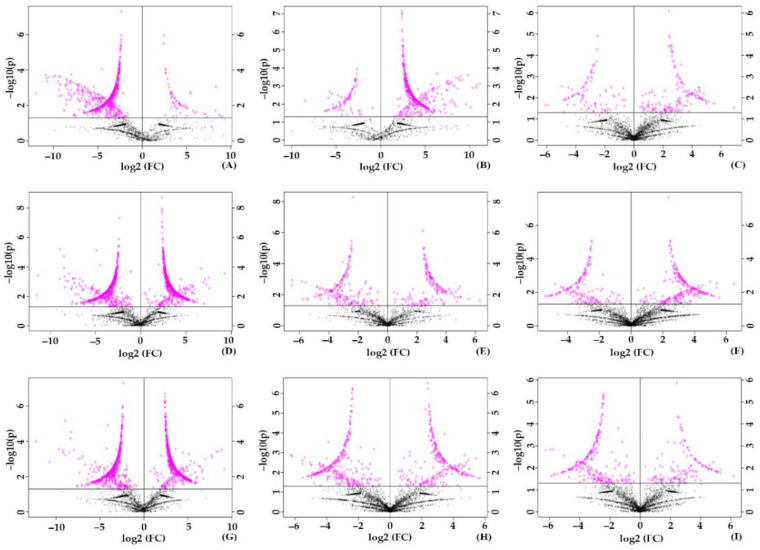
Volcano plot of significant metabolites during SA stress. (**A**) Control vs. treated_1. (**B**) Treated_1 vs. treated_2. (**C**) Treated_2 vs. treated_3. (**D**) Control vs. treated_4. (**E**) Treated_4 vs. treated_5. (**F**) Treated_5 vs. treated_6. (**G**) Control vs. treated_7. (**H**) Treated_7 vs. treated_8. (**I**) Treated_8 vs. treated_9.

The comparison between groups revealed the level of production in each unique and common metabolite. Metabolites had a threshold fold change of one and, with a *p*-value of 0.05 in the t-test, was considered and depicted in volcano plots. In treated_1 vs. control, around 59 were upregulated, and 647 were downregulated (Figure 6A). Treated_2 has 467 upregulated metabolites than treated_1 (Figure 6B), while treated_3 has 87 upregulated metabolites than treated_2 (Figure 6C).

In 50 mM SA, in comparison with the control, treated_4 showed 632 upregulated metabolites and 589 downregulated metabolites (Figure 6D). Upon comparison between the treated groups, treated_5 has 315 upregulated and 234 downregulated metabolites against treated_4 (Figure 6E). Treated_6 has 268 upregulated and 177 downregulated metabolites against treated_5 (Figure 6F). Even though the upregulation of metabolite was prominent in 50 mM SA, the gradual decrease of metabolite expression signifies the effect of stress on seedlings.

In 100 mM SA stress, compared with control, treated_7 showed 684 upregulating and 610 downregulated metabolite factors (Figure 6G). A comparison between the treated group showed higher numbers of downregulated metabolites than upregulated ones. Analysis of treated_8 with treated_7 showed 267 upregulated metabolites, and the number of downregulated metabolites was 307 (Figure 6H). About 144 metabolites upregulated against 256 downregulated metabolite factors were shown during the comparison between treated_9 and treated_8 (Figure 6I). The trend in significantly expressed metabolites towards a steep decrease highly suggests the effect of stress on seedlings. One hundred mM SA was highly stressful to plants, and its detrimental effect was visible by the high downregulated metabolites.

### 2.4. Orthogonal Partial Least Square Discriminant Analysis (OPLS-DA) on the Validation of Significant Metabolites in Stressed Groups

Each experimental group was separated based on the OPLS-DA in metabolites. The S-plot represented the pictorial depiction of metabolites, and those outside the red box were significantly expressed (Supplementary File S1). The R2X value obtained was the observed value, whereas Q2 was the expected OPLS-DA value. The closer observed to the anticipated, the higher the model’s fit. Based on the R2X value in P1, the stressed groups were separated. The R2X value in the q1 component was the basis for the separation of individuals within each group. The percentage R2X value in P1 was known as the T score, whereas the percentage value of R2X in Q1 was the orthogonal T score. These values made a pictorial depiction of stressed groups and members inside each group.

**Figure 7:**
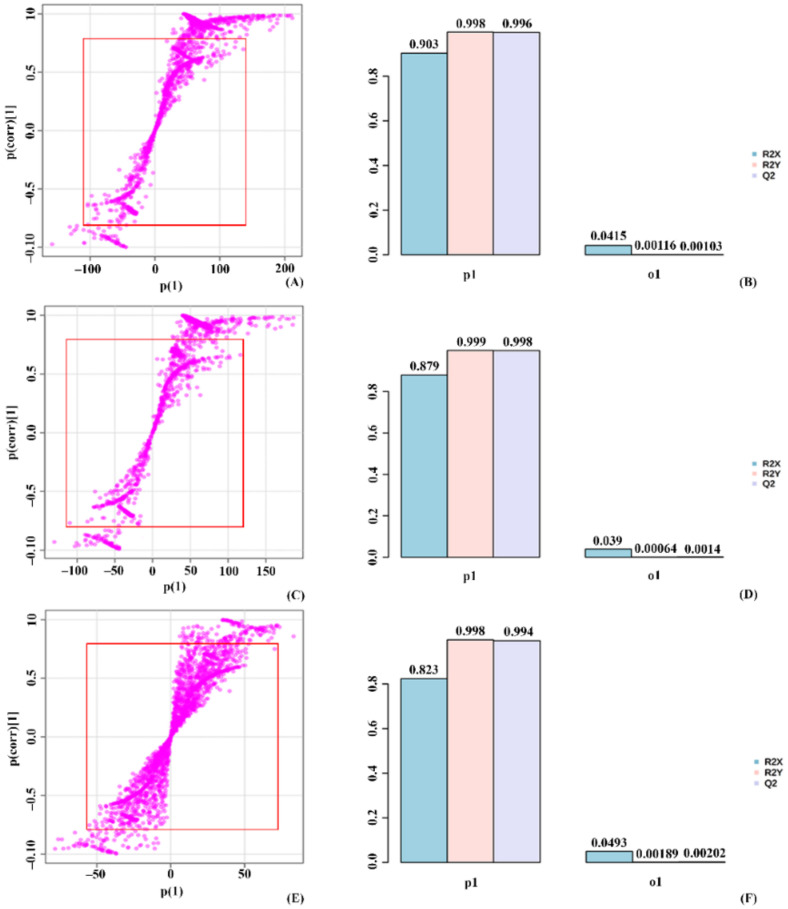
OPLS-DA plot grouping 10 mM stressed plants based on significantly expressed metabolites. (**A**) S-plot for control vs. treated_1, (**B**) mdsl plot with T-score and orthogonal T-score for control vs. treated_1, (**C**) S-plot for treated_1 vs. treated_2, (**D**) mdsl plot with T-score and orthogonal T-score for treated_1 vs. treated_2, (**E**) S-plot for treated_2 vs. treated_3, (**F**) mdsl plot with T-score and orthogonal T-score for treated_2 vs. treated_3.

OPLS-DA of the control and treated_1 group showed a T score of 90.3% and an orthogonal T score of 4.15% (Supplementary File S2). The significant metabolites used for the analysis were represented outside the S-plot red box. The mdsl plot showed the expected R2X and observed R2X value for control and treated_1. The separation of treated_2 with treated_1 had a T score value of 87.9% and an orthogonal T score of 3.9%. S-plot showed the significant metabolite used for the study, and the mdsl plot has the expected and observed R2X value used in determining the T score. In the case of treated_2 and treated_3, the T score value in the group’s separation was 82.3%. The individuals in each group were separated with an orthogonal T score of 4.9%. The mdsl plot with the expected and observed R2X value used in determining the T score value shown in the metabolites used for calculation are represented in the S-plot (Figure 7).

**Figure 8:**
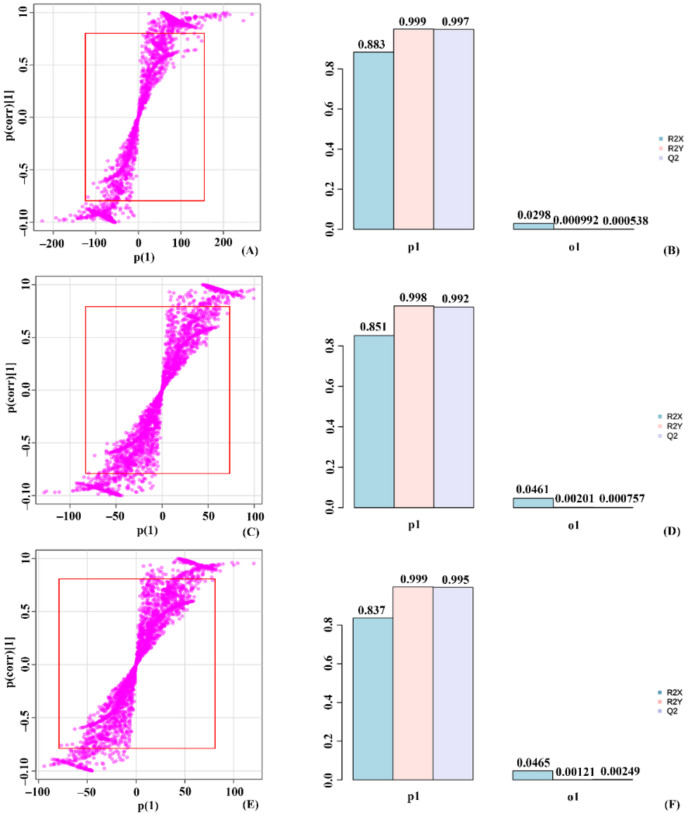
OPLS-DA plot grouping 50 mM stressed plants based on significantly expressed metabolites. (**A**) S-plot for control vs. treated_4, (**B**) mdsl plot with T-score and orthogonal T-score for control vs. treated_4, (**C**) S-plot for treated_4 vs. treated_5, (**D**) mdsl plot with T-score and orthogonal T-score for treated_4 vs. treated_5, (**E**) S-plot for treated_5 vs. treated_6, (**F**) mdsl plot with T-score and orthogonal T-score for treated_5 vs. treated_6.

In the 50 mM SA stressed condition, control vs. treated_4 showed a T score of 88.3% and an orthogonal T score of 2.93% during the separation (Supplementary File S2). The mdsl plot indicated the expected and observed R2X value of significant metabolites shown in the S-Plot. Treated_4 and treated_5 were separated with about a 85.1% T score value and an orthogonal T score value of 4.61%. The significant metabolites used for the study were depicted in the S-plot, and the mdsl plot shows the expected and observed R2X scores. An approximate 83.7% T score value and 4.65% orthogonal T score, treated_5, and treated_6, were plotted. The significant metabolites used to determine these values were depicted in the S-plot, whereas the mdsl plot shows the observed and expected R2X value (Figure 8).

**Figure 9:**
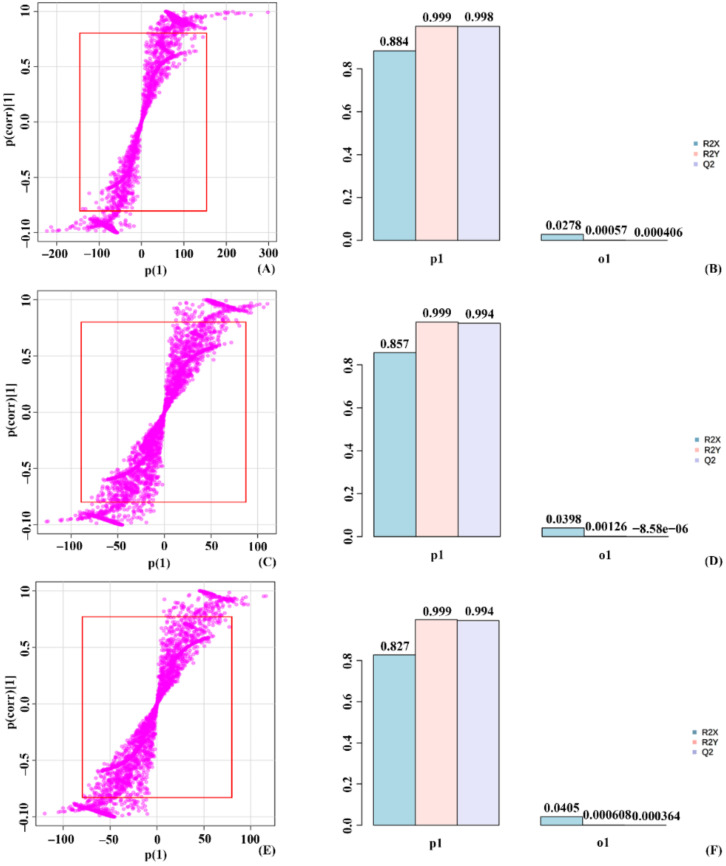
OPLS-DA plot grouping 100 mM stressed plants based on significantly expressed metabolites. (**A**) S-plot for control vs. treated_7, (**B**) mdsl plot with T-score and orthogonal T-score for control vs. treated_7, (**C**) S-plot for treated_7 vs. treated_8, (**D**) mdsl plot with T-score and orthogonal T-score for treated_7 vs. treated_8, (**E**) S-plot for treated_8 vs. treated_9, (**F**) mdsl plot with T-score and orthogonal T-score for treated_8 vs. treated_9.

In a 100 mM stress condition, control and treated_7 showed a T score of 88.4% and an orthogonal T score of 2.78% upon separation in the score plot (Supplementary File S2). The significant metabolites used for the analysis were represented outside the S-plot red box. The mdsl plot showed the expected R2X and observed R2X value for control and treated_7. The separation of treated_7 with treated_8 was with a T score value of 85.4% and an orthogonal T score of 4.15%. The S-plot showed the significant metabolite used for the study, and the mdsl plot has the expected and observed R2X value used in determining the T score. With an 85.7% T score value and 3.98% orthogonal T score, treated_8 and treated_9 were plotted. The significant metabolites used to determine these values were depicted in the S-plot, whereas the mdsl plot shows the observed and expected R2X value (Figure 9).

### 2.5. Correlation of Significantly Active Metabolites in the Stressed Condition

**Figure 10:**
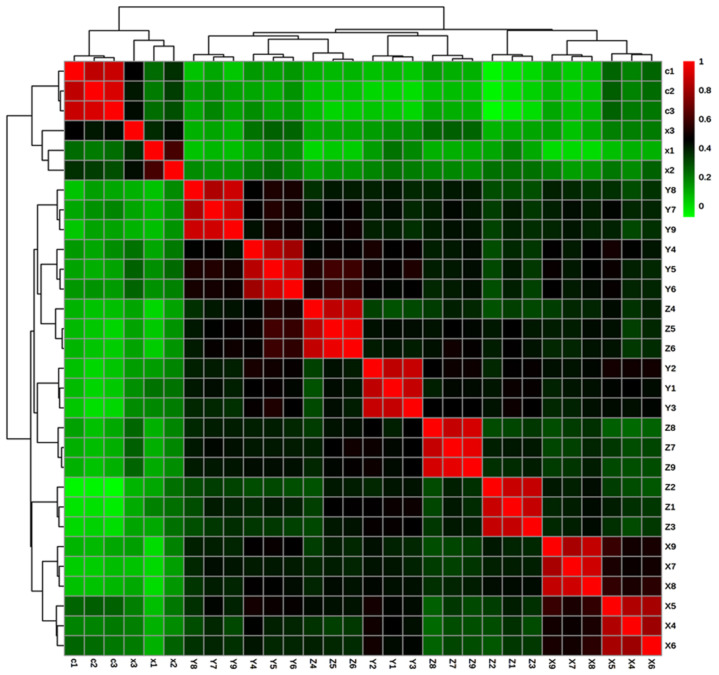
Correlation plot with dendrogram showing the grouping of SA stressed seedlings using Pearson’s correlation.

Pearson correlation between all the stressed groups showed a positive correlation (Figure 10). The significant metabolites were used for correlation and correlation values were given in Supplementary File S3. Individuals within the group established a correlation coefficient around one. This high correlation indicates specific stress on metabolites’ production in the individuals of the same group. Comparison with different groups revealed that control and treated_1 showed a lesser correlation with all other groups. The correlation coefficient of treated_1 was below 0.2 with all other groups, indicating that the effect of low concentration of SA did not produce any stress during the initial stages.

Similarly, treated_4 (Y1, Y2, Y3) and treated_7 (Z1, Z2, Z3) have the same correlation coefficient. It indicates that the initial stages showed a lesser effect on metabolite expression apart from SA concentration. All other groups have a comparatively higher correlation coefficient with each other. From these correlation coefficient values, it is clear that metabolites’ production during SA concentration was almost similar, and only the expression level varied for these metabolites.

The relationship between the stress group’s effect, a hierarchical cluster, was constructed using the Pearson correlation matrix and separated using Ward’s method. Two main branches were formed on the tree, with the first one containing control and treated_1, while the other branch was again divided into two. The small sub-branch consisted of treated_4 and treated_7, while the remaining stressed groups were in the other sub-branch. Treated_6 and treated_8 were plotted far away from the tree’s control, indicating that they were the most affected stress groups. Treated_5 and treated_9 were seen closed together in a small cluster due to the higher effect of 100 mM SA. On the 6th day, 100 mM SA might become toxic to the plants, resulting in lesser metabolites expression. Based on this lesser number and expression, treated_5 and treated_9 were grouped in the same small cluster.

### 2.6. Principle Component Analysis and Partial Least Square Discriminant Analysis of Significant Metabolites in the Classification of Experimental Groups

**Figure 11:**
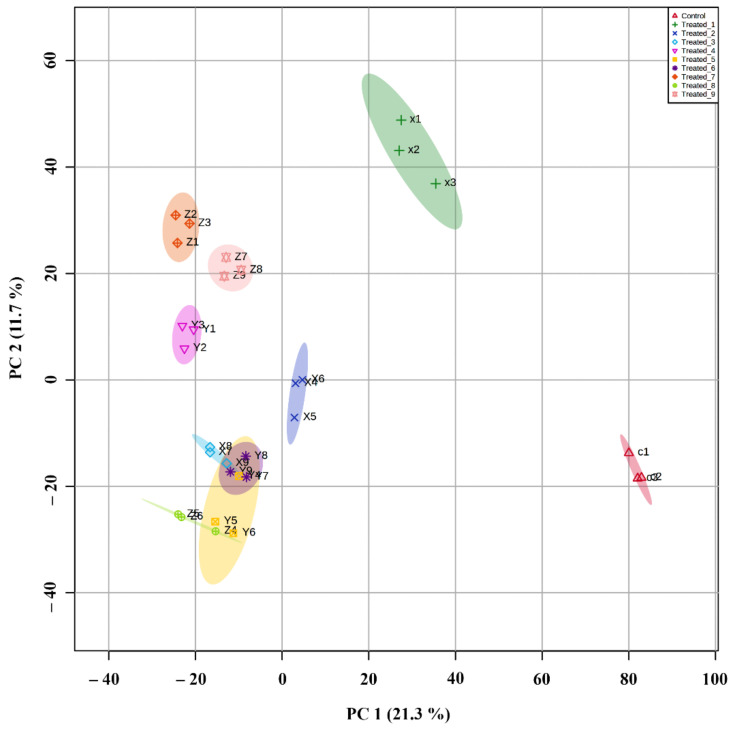
PCA analysis between the significantly produced metabolites and a plot showing the separation of the stressed group.

Based on the PCA of significant metabolites, the stressed groups separated based on PC1 of 21.3% and PC2 of 11.7%. The control group was the one placed the farthest in the score plot, followed by treated_1. All other groups were separated into two clusters in which treated_4, treated_7, and treated_9 were in one set and others in the next. In the third group, treated_3, treated_5, treated_6, and treated_8 were closer to each other. They indicated the production and expression of similar metabolites during the progression of stress (Figure 11).

**Figure 12:**
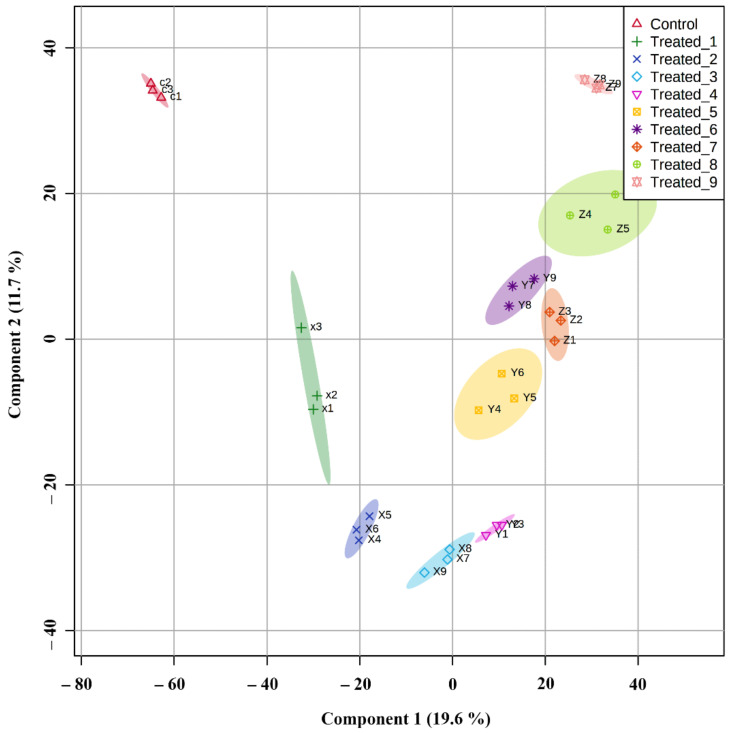
PLS-DA analysis on significantly expressed metabolites allows for grouping of stressed seedlings.

In the PLS-DA of significant metabolites, the groups were separated in a refined manner. Control was seen in a separate group where all the treated groups were seen closely with component 1 value of 19.6% and component 2 value of 11.7%. Treated_3 and treated_4 were closer in the plot, indicating that 10 mM SA’s effect on the 6th day was similar in production and expression of metabolite in 50 mM SA on the 2nd day. The arrangement of treated groups with 10 mM SA stresses was placed separately in the plot and validated the lower concentration’s mild effect. Fifty mM was more effective in stress generation with minimal lethality, which is evident with the arrangement in the middle of the treated groups. Treated_9 was seen far out in the plot, suggesting the lethality of 100 mM SA on the 6th day. The lethality was visible in the morphology of the plants, as mentioned earlier, and substantiated by the arrangement of treated_8 near treated_6 (Figure 12).

### 2.7. Determination of Metabolic Pathways Activated during Stressed Conditions

**Figure 13:**
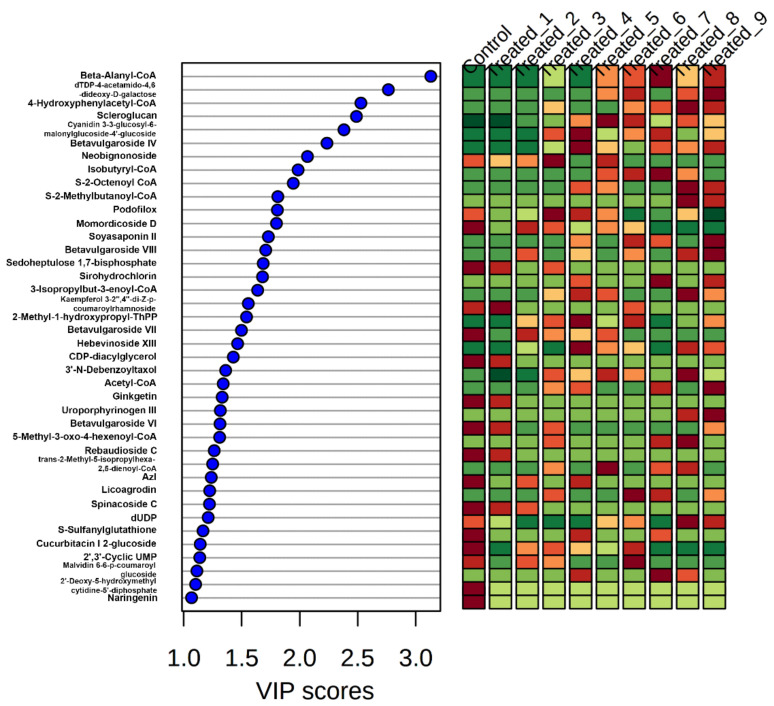
VIP scores of significantly expressed metabolites were identified with PLS-DA analysis, which was adopted for pathway identification.

From the Metlin library search data, various compounds in the significant metabolite groups were identified. With PLS-DA, the VIP score of these metabolites was revealed. The metabolites with a VIP score over one are shown in Figure 13. These compounds were significantly attributed to the responses of plants towards stress conditions and can be treated as putative marker compounds. These compounds belong to phenols, flavonoids, and terpenoids, and those of intermediated compounds in pathways were expressed differentially during various SA concentrations. For instance, the intermediate compounds of various pathways in fatty acid metabolism and shikimic acid pathway and terpenoid synthesis pathway were expressed more during the initial period of stress induction. The intermediate Beta-Alanyl-CoA showed a 24-fold increment than the control condition in treated_7. Isobutyryl-CoA, S-2-Octenoyl CoA, 5-Methyl -3 –oxo -4 -hexenoyl -CoA, 4-Hydroxyphenylacetyl -CoA, 3-Isopropylbut- 3-enoyl -CoA, 5-Methyl-3-oxo-4-hexenoyl-CoA, trans- 2-Methyl- 5-isopropylhexa- 2,5- dienoyl- CoA, etc., showed more than 20-fold expression than that of the control and was observed in higher concentration and a later time period of stress condition. All these fatty acid metabolic intermediates were expressed in treated_3, treated_7, and treated_8, whereas only Isobutyryl-CoA and trans- 2-Methyl- 5-isopropylhexa- 2,5- dienoyl- CoA was expressed in treated_5 samples. While observing the putative metabolites detected from different classes like alkaloids, terpenoids, flavonoids, and phenols, it is evident that 50 mM stressed conditions produced a maximum number of metabolites. Contradictory to intermediated compound expression levels, most alkaloids, phenolics, flavonoids, terpenoids, and lignin compounds were high in stressed groups with lower or no expression level of intermediate compounds. For instance, the expression level of putative alkaloid 3′ 4′ anhydrovinblastin in treated_2 was 20-fold higher than that of the control and a lesser expression was observed in all other treated groups. In the case of flavonoids, Kaempferol 3-(2′′,4′′-di-(Z)-p-coumaroylrhamnoside) was expressed in treated_1 and treated_6 with a fold change over 18 from treated_7, 8, and 9. Similarly, licogordin, Cyanidin 3-(3-glucosyl-6-malonylglucoside)-4′-glucoside also showed over a 2-fold increase in treated_5,and 6 with that of treated_7, 8, and 9. The expression level of the putative terpenoids compounds like betavulgaroside (18-fold), asiaticoside (17-fold), and mubenin B (20-fold) were higher in treated_6 samples compared with the control and similarly, the expression level of deslanoside (22-fold), momordicoside (13-fold), hebevinoside (15-fold), notoginsenoside H (18-fold), and soyasaponin A (14-fold) in treated_3 compared with the control. However, these terpenoids showed a 2- to 10-fold increase from that of treated_7, 8, and 9. The detailed expression level of each significant metabolite and those with VIP values over 1 were provided in Supplementary File S4.

**Figure 14:**
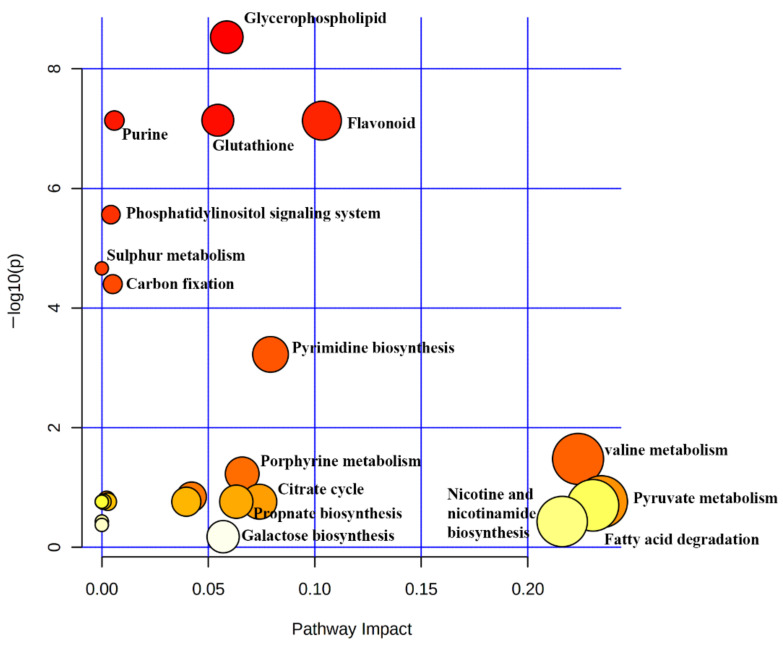
Graph showing differential production of metabolites pathways on the onset of SA stress.

These significant metabolites were used to identify various pathways expressed during the stress and around 29 metabolic pathways became expressed (Figure 14). Around 27 intermediated compounds were identified during the stressed periods. Some of the individual intermediate compounds were responsible for activating multiple pathways. On the other hand, various intermediates of the same metabolic pathways were detected, in which valine, leucine, and isoleucine degradation pathways showed maximum intermediated compounds up to six.

The intermediate metabolites responsible for activating multiple pathways were CDP-diacyl glycerol, luteolin, Acetyl CoA, and NAD^+^. Acetyl CoA was the primary, intermediate compound responsible for activating 15 metabolic pathways. Acetyl CoA was activated in treated_3, treated_4, treated_7, and treated_9, in which the highest expression was in treated_7. Comparison with the secondary metabolites confirmed the role of acetyl CoA in activating these pathways. Terpenoids were the primary group of secondary metabolites expressed in the stressed groups. A differential expression of 51 putative terpenoids was identified during the SA stress (Supplementary File S4). Of these terpenoids, 31 were upregulated in one or a few treated groups, while the others were downregulated.

Flavonoids and phenolic compounds show activation and upregulation in all of the stressed groups (Supplementary File S4). Flavonoids and phenolic metabolites were activated in higher numbers in treated_1 than in any other category of metabolites. Similarly, alkaloids and glycoside were also activated in treated_1, but the number was meager. Apart from the activation, all these metabolite groups tend to have higher expression in the 50 mM stressed group (treated_4, 5, and 6). In the case of 100 mM (treated_7, 8, and 9), peptides and compounds produced from chlorophyll degradation were found to be highly upregulated than any other metabolic fractions.

## 3. Discussion

Salicylic acid imparted different responses in the treatment group with the concentration. Lower concentrations showed a minimal response and good growth characteristics, while higher concentrations showed stress responses. Even though the morphology has visible changes during higher concentration, it was not seen during the initial stage. Treated_1, treated_4, and treated_7 have a similar morphological appearance with green and intact leaves. As the day progressed, treated_2 and treated_3 had similar morphological characters such as entire green leaves like that of the control and treated_1 plants, while treated_5 and treated_6 showed slight yellowing of the leaves. Treated_8 and treated_9 showed greater aberration compared to other groups with severe necrosis and leaf falling. The minor changes in their morphology during initial stress response and lower concentrations rule out the chance of toxicity by SA. Metabolomic analysis revealed a difference in pattern upon activating metabolites in all the stressed conditions. A comparison of each treated group with control revealed all activated metabolites. The least number of metabolites were activated at the lower concentration of SA, and as the concentration increased, more metabolites were activated. In *Zea mays*, SA treatment increased the production of secondary metabolites and biomass content in plants concerning foliar size increase and root growth [34], whereas, under drought conditions and SA stress, the secondary metabolite concentration increased [35]. These findings substantiate the dose-dependent action of SA upon the mediation of various stress.

The trend in upregulation and downregulation of significant metabolites concorded with the total number of metabolites identified. The higher number of upregulated metabolites in 50 mM indicated the optimal response of plants towards SA. The higher downregulated metabolite in 100 mM stated the severity of the stress. Similarly, metabolite production in various plants was regulated by SA. The production of essential oils in *Ocimum basilicum* [36], oleoresins in *Pinus* [37], and triterpenes in *Nigella* [38] were regulated by SA. In *Pharbitis nil* plants, the optimal concentration of SAs was used to induce flowering, and, at lower concentrations, the plants retained their normal physiological functions. Similar to *A. polystachya* seedling, higher SA concentration was toxic to the plant and delayed the flowering stage [39]. As SA stress increased in *Lactuca sativa*, the chlorophyll content decreased, whereas the carotenoid and proline content increased [40].

By correlation analysis, the comparison among the groups was identified. The groups with a higher Pearson correlation coefficient showed similarity among metabolites during the stress condition, and those having a lower correlation coefficient tend to have lesser similarities. Principle component analysis also showed a similar pattern. PLS-DA [41] control and treated groups were classified and showed three main clusters. The lower stressed group with initial stages came under one cluster, while the stressed group with later stages were clustered together. The treatment group containing a higher amount of SA was grouped in the third cluster. The VIP score in PLS-DA was used to determine the importance of significant metabolites, and those that have high potential are selected as markers for stress in *A. polystachya*. Cross-validation of multivariate analysis performed using PLSDA and significant metabolites in each group’s separation was determined with opslda [42]. The R2X and T1 score was helpful in the optimal separation of the groups.

The metabolic pathways expressed during the stress were validated with the differentially expressed secondary metabolites. The authenticity of identified metabolites using MS-based studies was the primary concern in determining the pathways. The study conducted in *Aphanizomenon flos-aquae* using NMR (nuclear magnetic resonance) spectroscopy and LC-MS detected uncommon metabolites. The MS predicted mass of these phosphorylated nucleoside compounds was similar to the actual mass, and various NMR techniques further validated the detected compound [43]. Similar to that, these metabolites predicted the nature of stress exerted on seedlings during SA stress. Primary metabolites and secondary metabolite compounds previously known to be activated in biotic and abiotic stress conditions were determined in these stressed groups. The primary metabolite sedoheptulose 1,7-bisphosphate was activated only in a lower concentration of SA in treated_1 and treated_3. In *Nicotiana tobacco* mutants, hybridizing *Arabidopsis* cDNA associated with sedoheptulose production increased growth and development [44].

Further investigation in tomato plants showed downregulation of sedoheptulose concentration during methyl jasmonate stress. The plants showed leaf senescence symptoms associated with abiotic stress like drought, salinity, and temperature [45]. A similar response was seen in *Aphanamixis* seedlings as sedoheptulose concentration decreased even in minimal stress conditions as time progressed. The majority of metabolites identified were secondary metabolites, and just like primary metabolites and intermediate compounds, some of them were previously studied in various biotic and abiotic stress conditions. Alkaloids were expressed in a lower concentration of SA, and previous studies revealed that alkaloids were positively associated with biotic stress. Anhydrovinblastine was found to be expressed in 10 mM stressed condition, and this terpenoid indole alkaloid was known for protecting plants from herbivores and pathogens. It causes a delayed growth rate in larvae, fungi, and microbes [46]. In *Catharanthus roseus* anhydrovinblastine was produced by the action of a peroxidase-like enzyme. These enzyme isoforms were known for their activation during plant defense responses for better adaptation. A higher expression of these compounds was seen in wound-induced studies. The peroxidase isoform was associated with auxin catabolism and hydrogen peroxide scavenging activities [47]. Another alkaloid found in SA stress was amaranthin, which was higher in *Amaranthus* plants during abiotic and biotic stress. A higher content of amaranthin was found in plants under herbivorous attacks. While the larval invasion increases, amaranthin and other pigments production increases. Removal of larval stress causes a decrease in the amaranthin and pigment contents [48].

The second group of highly activated compounds belonged to the flavonoid and phenolic classes. Flavonoids were well known for their action during biotic and abiotic stress. Luteolin was found to be expressed in seedlings under stress. Previous studies revealed that luteolin was well known for free radical scavenging activity during UV radiation stress in leguminous plants [49]. In the plant cell, flavonoids were stored in vacuoles. During stress conditions, proteins sequestrate the reactive oxygen species to vacuoles for their detoxification. It was believed that flavonoids help maintain the internal ROS homeotic balance [50]. Studies in tea plants showed that epicatechin and its analogs were higher in water-stressed conditions and were determined as tea plants’ metabolic markers under drought stress [51]. The epicatechin analog was found in higher quantities in medium and high stressed conditions in *Aphanamixis* seedlings, indicating a higher concentration of SA was inducing drought-related states.

Similarly, with temperature and drought stress, the anthocyanin content was found to be increased in plants. A similar effect was observed for the pigmented flavonoids in *Aphanamixis* seedlings, which induced higher SA concentration. Studies in *Arabidopsis* proved the effect of drought, temperature, and salinity on increased anthocyanin production and active ROS scavenging activities [52]. Apart from this, studies showed that isoflavonoids like Genistein 7-O-glucoside-6′′-malonate were highly expressed in plants under cold stress conditions [53]. Previous studies in *Arabidopsis* showed that SA concentration was lesser during cold stress. SA blocked the activation of cold regulating pathways, which was proved by the down regulation in *Aphanamixis* seedlings.

The major secondary metabolite component activated during the study was terpenoids. As flavonoids, some of the terpenoids identified in *Aphanamixis* seedlings have previously been well studied for their activity against abiotic stress. The terpenoid Asiaticoside was highly expressed in *Centella* species during temperature and dehydration stress [54]. In *liquorice* seedlings, drought stress-induced with PEG showed an increment of genes in the terpenoid backbone synthesis pathway. The Glycyrrhizinic acid content was higher in those seedlings as the stress period increased [55]. A similar effect was observed in Glycyrrhizinic acid content SA-induced seedlings at higher concentrations during the stress period.

Another terpenoid found in higher quantity was ginsenoside, which was observed in American *ginseng*. Ginsenoside is responsible for maintaining plant growth during drought conditions [56]. In *ginseng*, drought increases ginsenoside production by activating the methyl jasmonate pathway. The signaling pathway was determined by an elicitation study using methyl jasmonate [57]. SA and methyl jasmonate pathways are associated together, which might be the reason for the production of ginsenosides in *Aphanamixis* seedlings. Pathogen infection studies in ginseng revealed the significance of the SA pathway in ginsenoside production. As the association with pathogens tends to increase, the gene responsible for SA production was found to be increased along with ginsenoside [58]. Cucurbitacin was expressed higher in lower concentrations of SA, and in Cucurbitaceae plants, cucurbitacin was activated during pathogen response [59]. From the differential expression of metabolites mentioned above and other significantly expressed metabolites during the SA stress condition, it was evident that SA concentration was responsible for the ineffective tackling of a tree during adverse conditions. During lower levels of SA concentrations, the seedlings tend to produce more alkaloids and toxic metabolites, which are responsible for resistance towards pathogens and herbivory. The expression level of intermediate compounds also suggested that the pathway expression towards biotic stress response compounds production. As SA concentration increases, the response toward free radicle scavenger compound productions increases. The level of expression of these compounds in 50 mM SA concentration suggested these effects. Higher expression of flavonoids, like Kaempferol 3-(2′′,4′′-di-(Z)-p-coumaroylrhamnoside) licogordin, Cyanidin 3-(3-glucosyl-6-malonylglucoside)-4′-glucoside, and terpenoids betavulgaroside, asiaticoside, mubenin B, deslanoside, momordicoside, hebevinoside, and notoginsenoside H and soyasaponin A over the intermediate compounds suggested the effective activation response toward free radicle scavenging.

Compounds like Beta-Alanyl-CoA, Isobutyryl-CoA, S-2-Octenoyl CoA, 5-Methyl -3 –oxo -4 -hexenoyl -CoA, 4-Hydroxyphenylacetyl -CoA, 3-Isopropylbut- 3-enoyl -CoA, 5-Methyl-3-oxo-4-hexenoyl-CoA, trans- 2-Methyl- 5-isopropylhexa- 2,5- dienoyl- CoA, etc., in 100 mM concentration with lower levels of end products also proposed the termination of the pathway due to its response towards extreme stress condition. The level of chloroplast content in the 100 mM stressed samples substantiate it. SA is known for evoking the abscisic acid pathway, which results in senescence in plants. Similarly, during senescence, chloroplast contents like porphyrin, chlorophyll, etc., are sequestrated in the younger portion of plants and the parts tend to undergo apoptosis. The morphological changes of severe leaf fall and yellowing of seedlings during the extreme stress levels validate these. Lower metabolic concentration of flavonoids, terpenoids and allied secondary metabolite in 100 mM stressed conditions further evident the arrest of metabolic pathways during the stress condition. From this dose-dependent temporal study in *A. polystachya* seedlings, it was evident that SA functions in cross-talks between pathways that are responsible for biotic and abiotic stress. The concentration level of SA will be responsible for the activation of various pathways, determining the survival of trees species during different types of stress.

## 4. Materials and Methods

### 4.1. Stress Induction in Seedlings

Seeds were collected from mature tree stock (voucher herbarium accession number KUBH 8874) from the garden to maintain homogeneity. The seeds were washed and dried in the shade to remove dirt and moisture and stored at 4 °C in the seed bank. Approximately 25% of the collected seeds were taken for the study and the remaining were kept in the seed bank. Seeds were germinated by soaking in acid-free paper and after two weeks, the seedlings were washed with 1% Bavestin (Carbendazim 50%, BASF, Chennai India Ltd. India), rinsed thoroughly in distilled water and transferred to planton jars (Tarson, India) containing autoclaved (120 °C, 15 psi, 18 min) Knops medium (Himedia, Mumbai, India). The planton jars were kept in the culture room with a controlled temperature and humidity (22 ± 2 °C, 50–60% humidity, 12 h photoperiod with 50–60 µεm-2s-1 light intensity). After two months, these seedlings were transferred to Knop solution supplemented with SA (Sigma Aldrich, Bengaluru, India) at 10 mM, 50 mM, and 100 mM concentrations for stress induction and were kept for 6 days.

### 4.2. Sample Harvesting and Extraction

For the metabolomics studies, specific parameters were followed in sample preparations [60]. A triplicate of samples was preferred for metabolomics studies to avoid biological variance. Instant freezing of samples after harvesting was preferred to snap shut the expression of tissues. One hundred mg of plant tissue was taken and ground to a fine powder using liquid nitrogen. The powder was extracted in 1 mL HPLC (High-Performance Liquid Chromatography) grade ice-cold methanol (Merck, Darmstadt, Germany) to avoid redox reaction on compounds. Extracts were centrifuged, pellets were discarded, and the supernatant was used for further analysis.

### 4.3. Parameters Used for LC-MS Analysis

Extracts were analyzed in LC-MS/MS 8045 triple quadrupole ESI (Electron Spray Ionization) system (Shimadzu, Kyoto, Japan). Acetonitrile (Merck, Darmstadt, Germany) and 1% formic acid (Merck, Darmstadt, Germany) in water (Merck, Darmstadt, Germany) was used as the solvent system. The extract was passed through a C18 column at a temperature of 40 °C by a gradient LC time program of 40% acetonitrile to 100% and back to 40% in a linear gradient for 35 min. Each compound in the extract was detected using MS through electron spray ionization. The compounds were charged at 70 ev in the ESI probe, passed through the collision cell, and caught in quadrupole 1. Nitrogen was used as the carrier gas, and Argon (99.999%) was used as collision gas for the system. Every solvent used for the study was of LC-MS grade to avoid contamination of any kind.

### 4.4. Processing of Raw Data using XCMS

Metabolic profiling of stressed plants was carried out using XCMS online software (version 3.7.1, Siuzdak Lab at Scripps Research, San Diego, CA, USA) [61,62]. Raw data were obtained from LC-MS analysis in the format of .cdf uploaded to the XCMS server and peak determination was carried out between groups. Based on the M/Z (Mass/Charge) ratio of each peak, compounds were identified using the Metlin (scripps research) [63] database search. The intensity of each peak was analyzed and used for further studies.

### 4.5. Statistical and Pathway Enrichment Studies using Metaboanalyst

The enriched data obtained after XCMS analysis was used for determining differential expression and pathway analysis using metaboanalyst [64]. T-test followed by fold change analysis was performed to determine significantly expressed metabolites and differential expression of metabolites in two experimental groups. The effectiveness of significant metabolites in the separation of treatment was determined by orthogonal partial least square discriminant analysis (OPLS-DA). Classification of stressed groups done by Pearson correlation of all groups followed by principal component analysis (PCA) and partial least square discriminant analysis (PLS-DA). The significant metabolites with VIP scores over one were considered for pathway determination studies.

## Data Availability

The data presented in this study are available upon request from the corresponding author.

## Supplementary Materials

The following supporting information can be downloaded at: https://www.mdpi.com/article/10.3390/molecules27092966/s1, Supplementary File S1: Significantly expressed putative compounds with mass and intensities during SA stress in *Aphanamixis polystachya* seedlings, Supplementary File S2: OPLSDA plot of SA stressed *Aphanamixis* seedlings, Supplementary File S3: Correlation matrix of significantly expressed metabolites during SA stress condition, Supplementary File S4: Box plot showing differentially expressed metabolites during the SA stress condition.
