# Supplementary material for: Untargeted Metabolomic Approach to Determine the Regulatory Pathways on Salicylic Acid-Mediated Stress Response in Aphanamixis polystachya Seedlings
Source: Molecules. 2022 May 6;27(9):2966. doi: 10.3390/molecules27092966 (PMC9102903; doi:10.3390/molecules27092966)
Supplement: Supplementary file 1 [file molecules-27-02966-s001.zip › Supplementary files/Supplementary file S2 OPLSDA plot of SA stressed Aphanamixis seedlings.pdf]

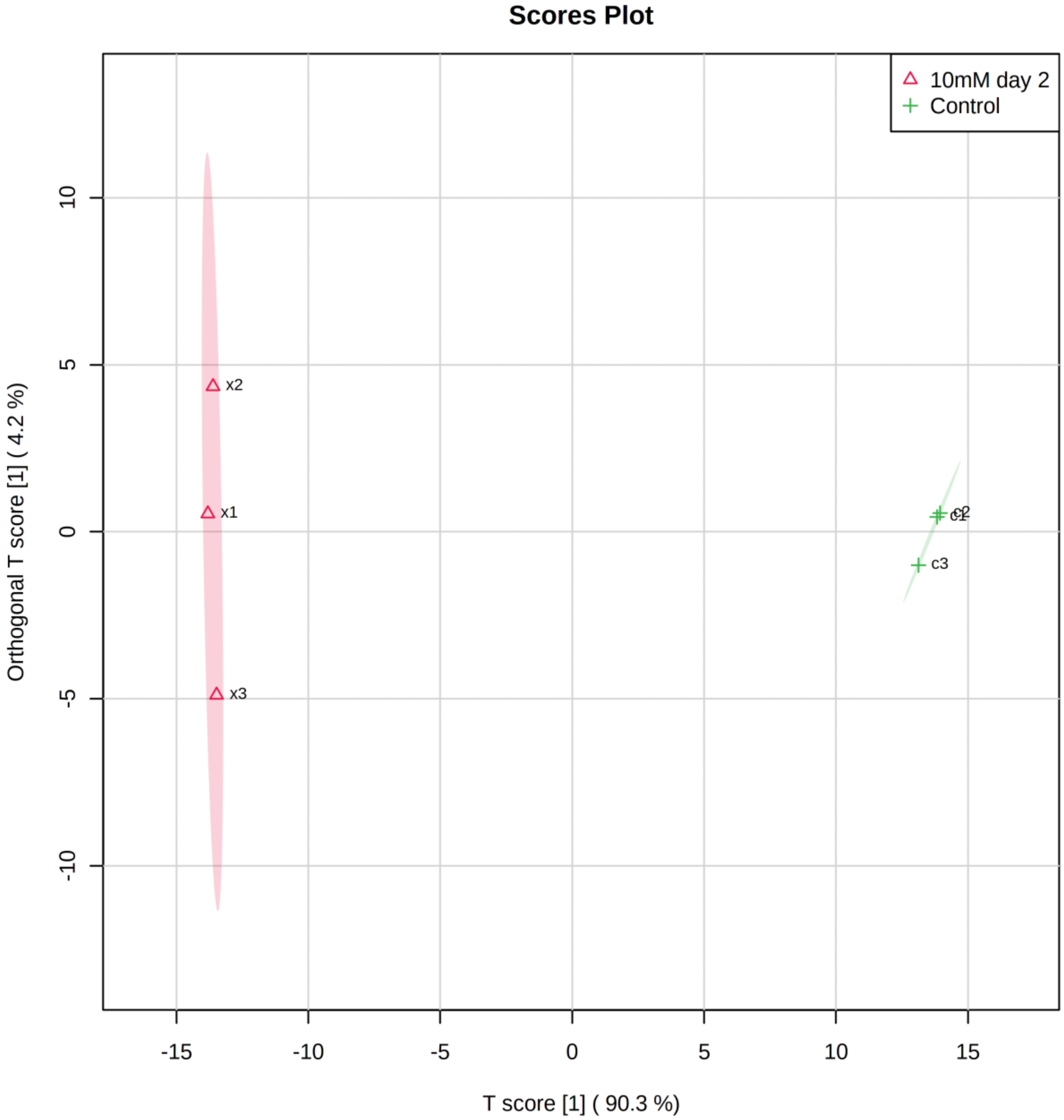

Figure S1. OPLSDA plot for control vs Treated_1 samples of *Aphanamixis polystachya* seedlings

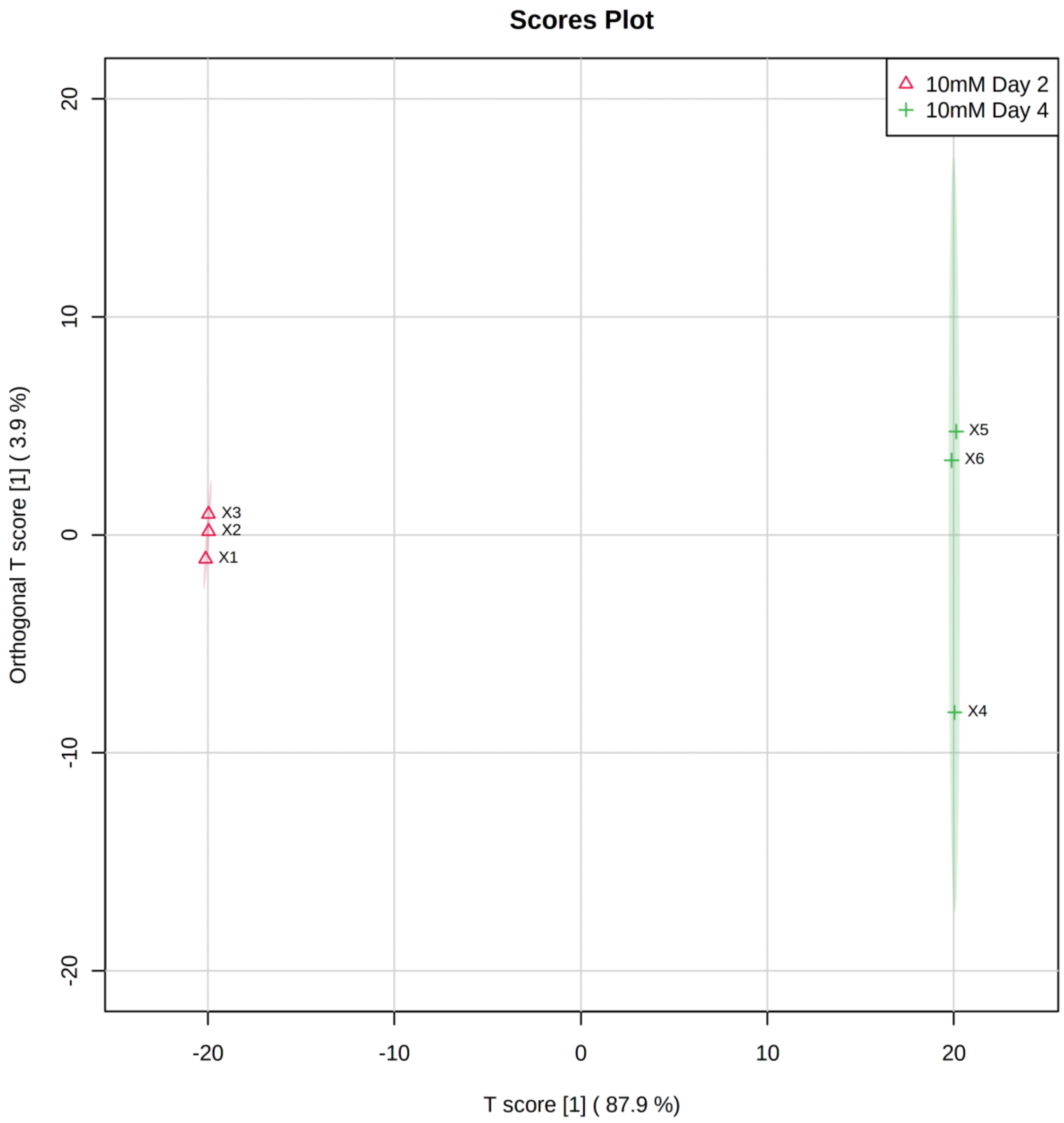

Figure S2. OPLSDA plot for Treated_1 vs Treated_2 samples of *A. polystachya* seedlings

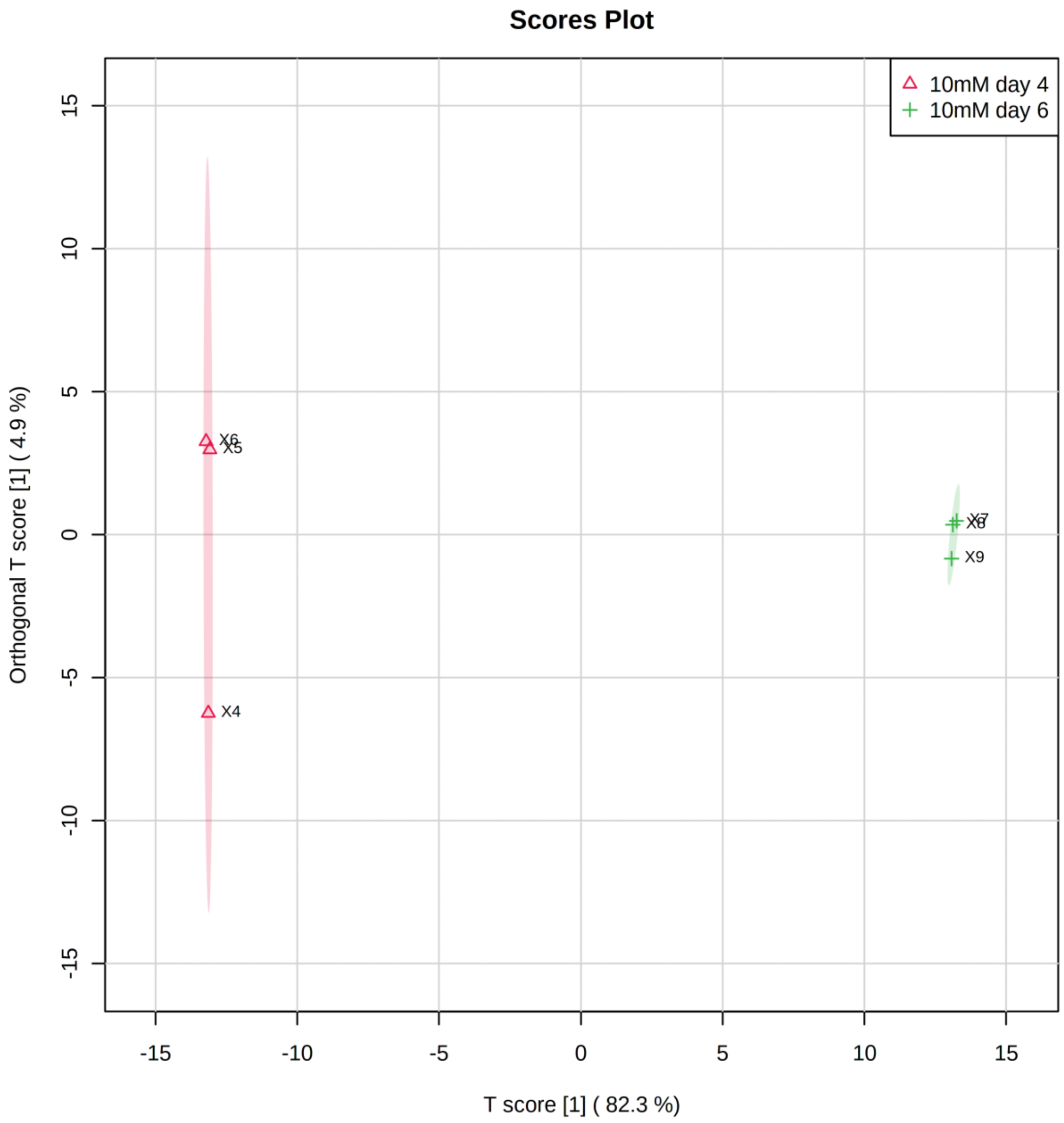

Figure S3. OPLSDA plot for Treated_2 vs Treated_3 samples of *A. polystachya* seedlings

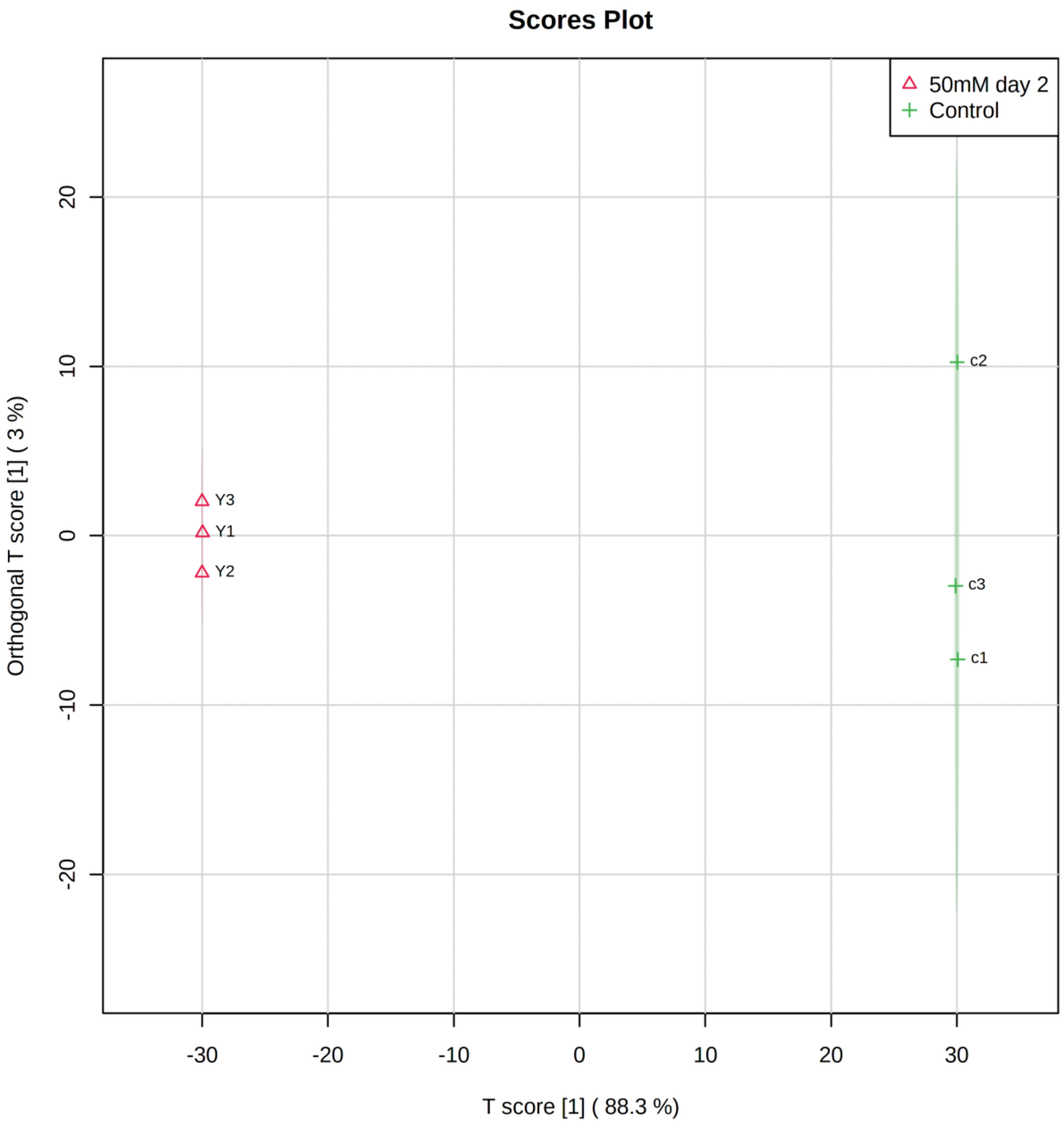

Figure S4. OPLSDA plot for Control vs Treated_4 samples of *A. polystachya* seedlings

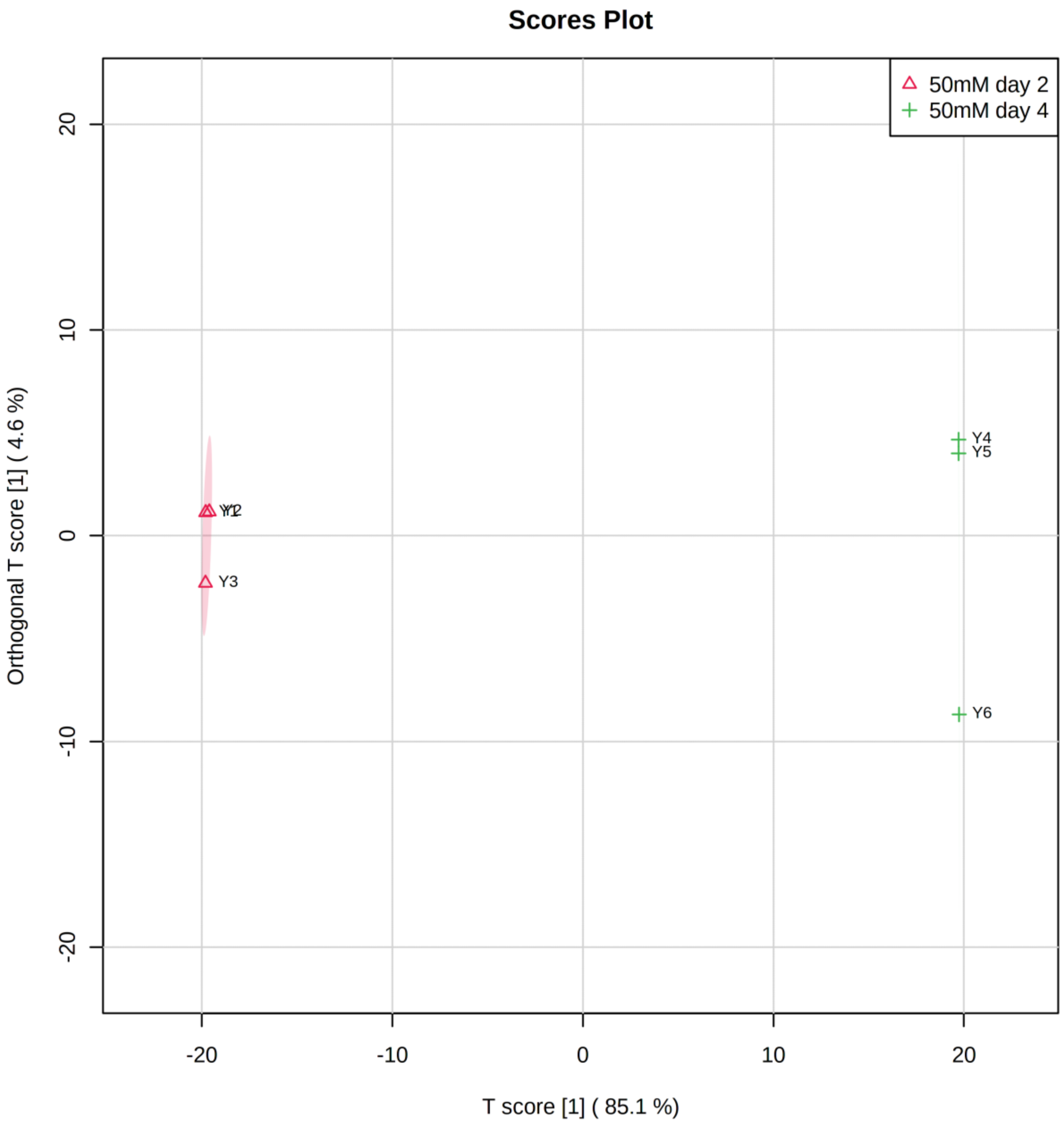

Figure S5. OPLSDA plot for Treated_4 vs Treated_5 samples of *A. polystachya* seedlings

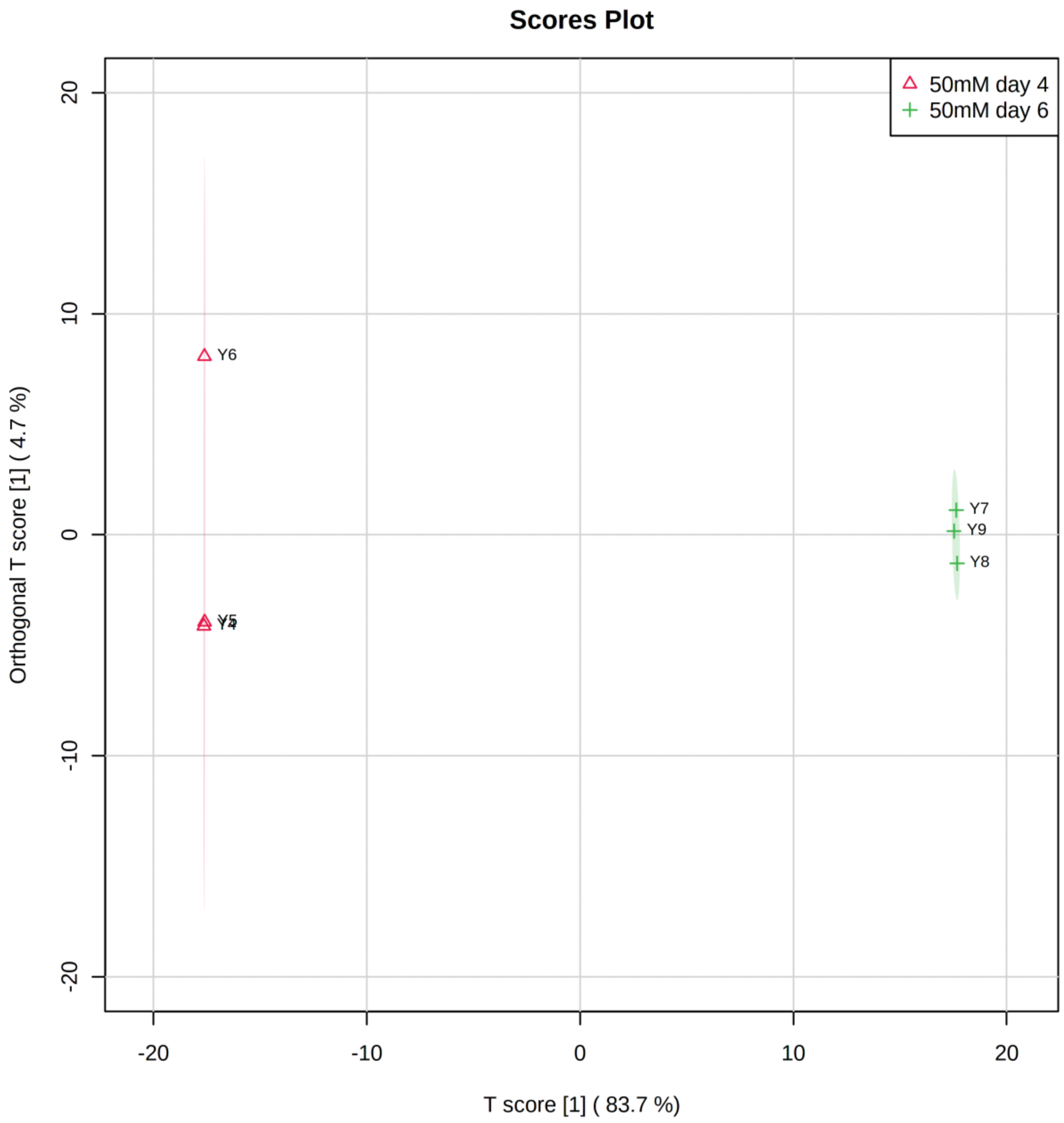

Figure S6. OPLSDA plot for Treated_5 vs Treated_6 samples of *A. polystachya* seedlings

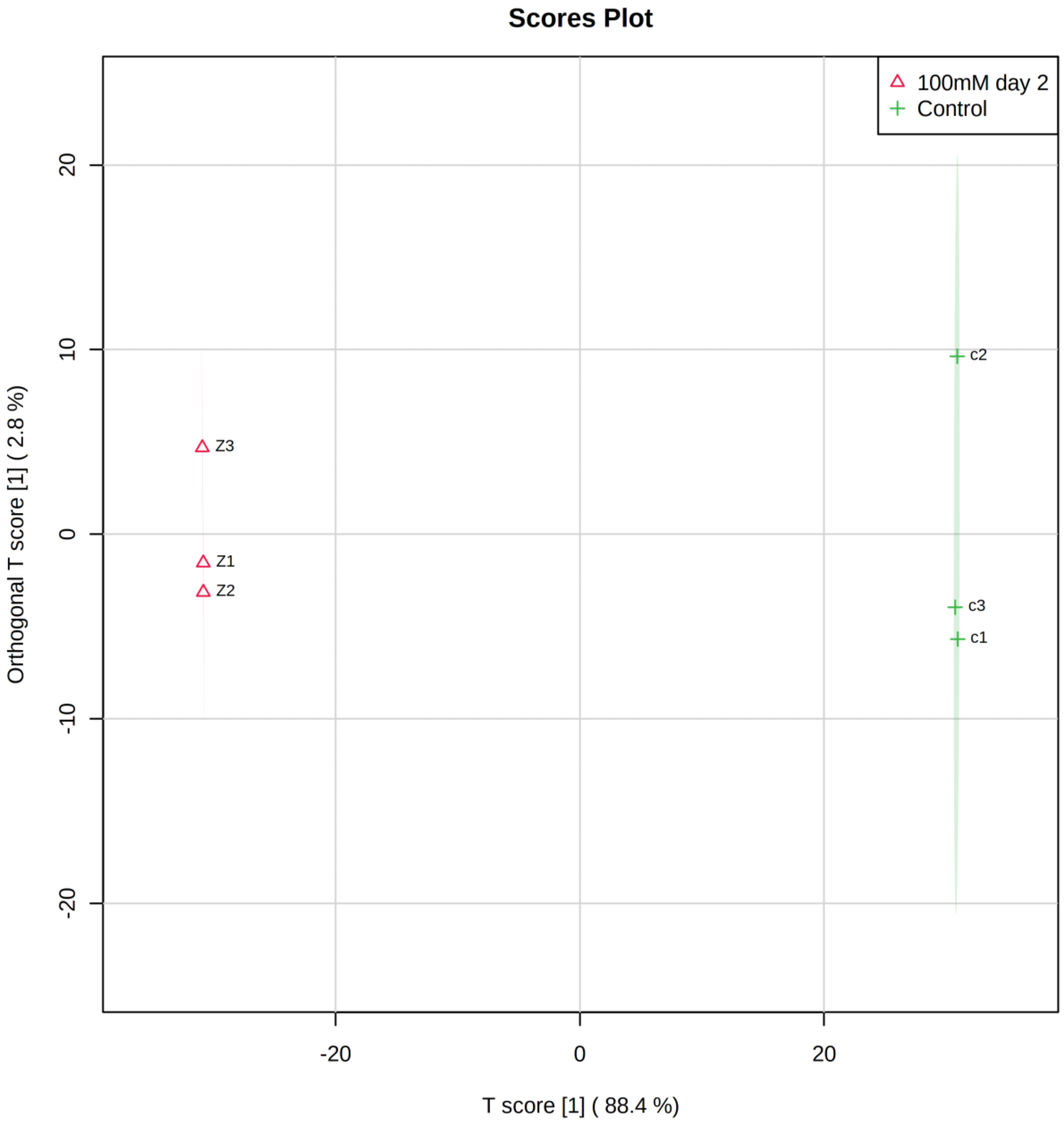

Figure S7. OPLSDA plot for Control vs Treated_7 samples of *A. polystachya* seedlings

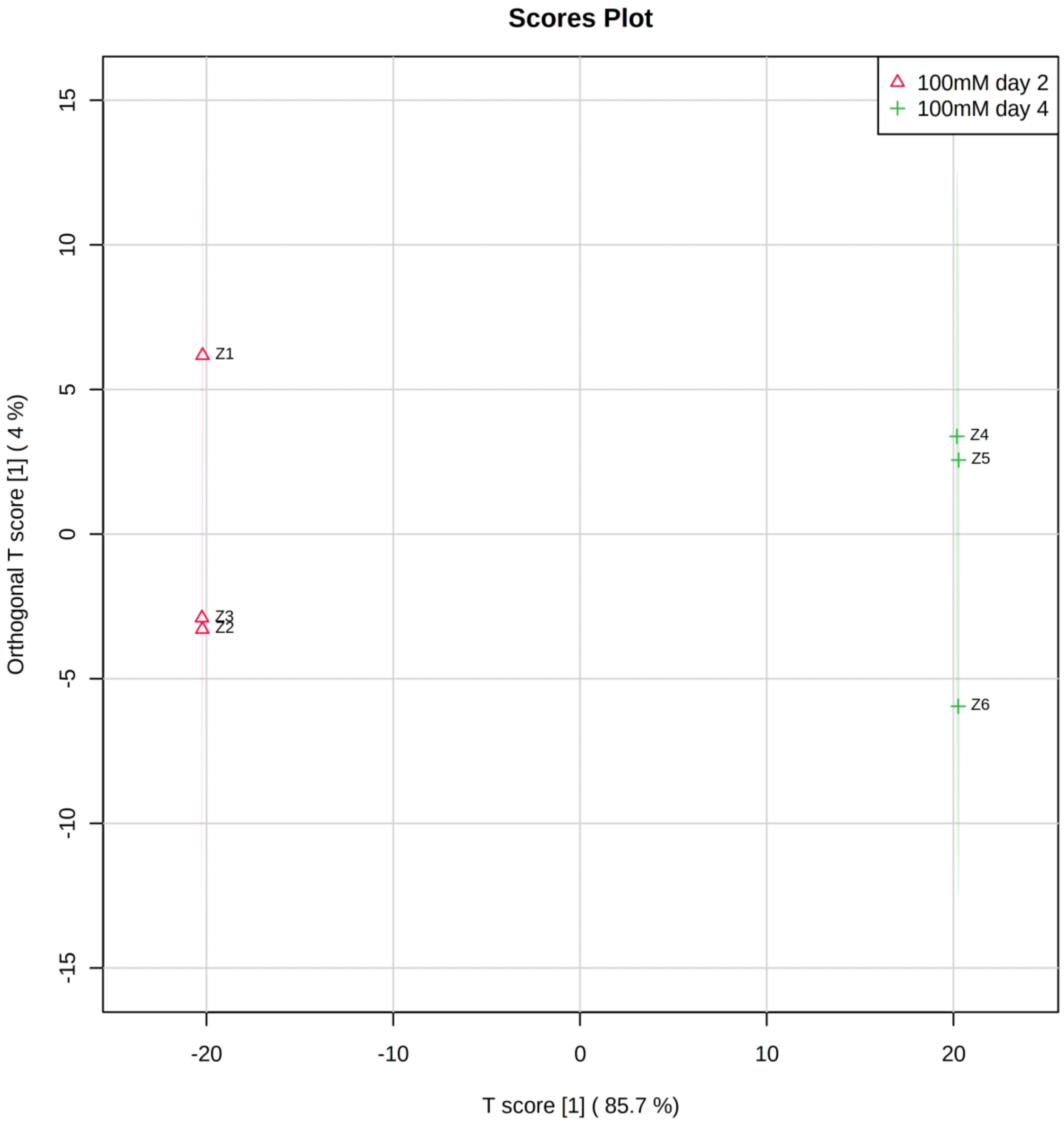

Figure S8. OPLSDA plot for Treated_7 vs Treated_8 samples of *A. polystachya* seedlings

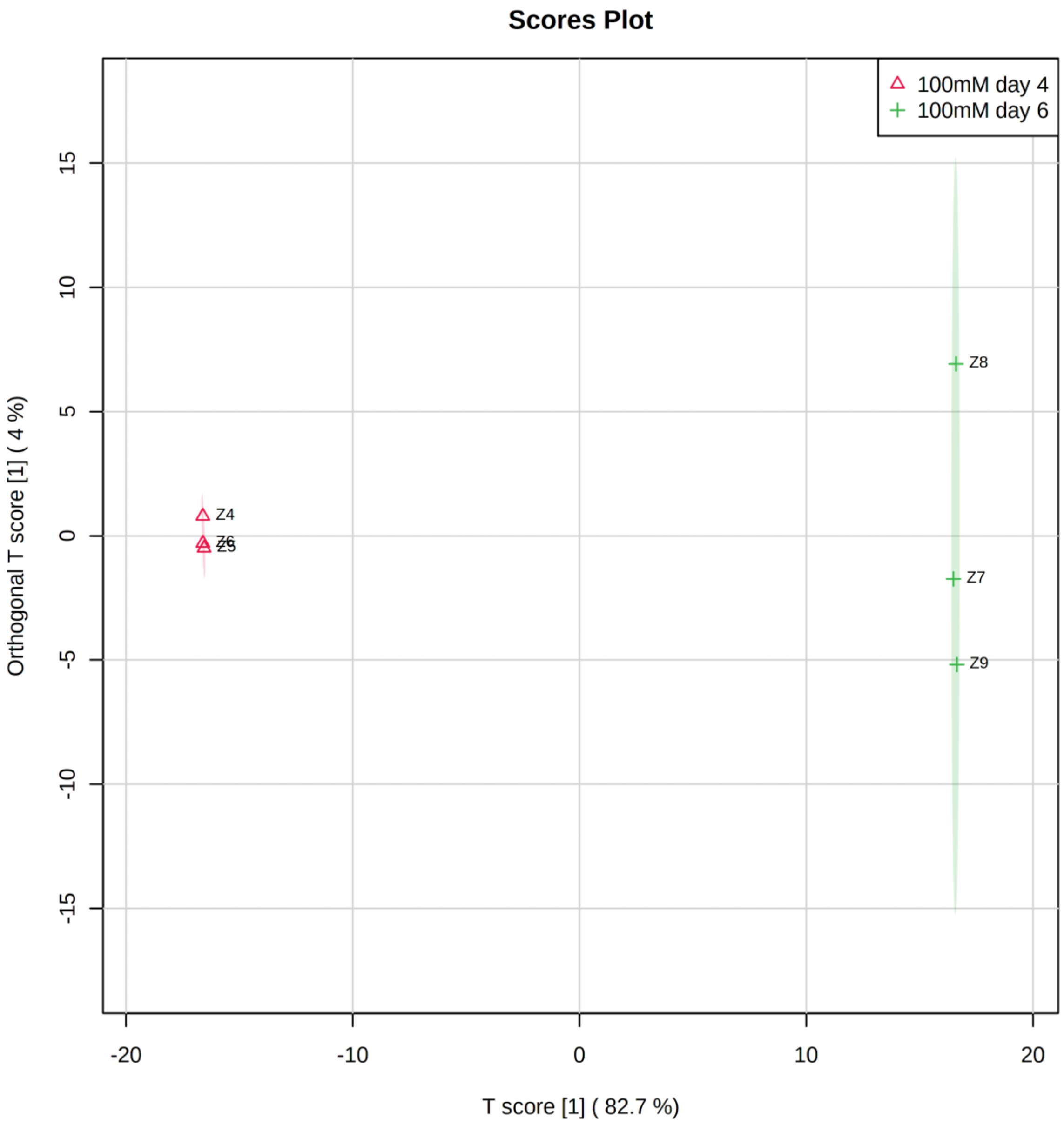

Figure S9. OPLSDA plot for Treated_8 vs Treated_9 samples of *A. polystachya* seedlings
