# Supplementary material for: Untargeted Metabolomic Approach to Determine the Regulatory Pathways on Salicylic Acid-Mediated Stress Response in Aphanamixis polystachya Seedlings
Source: Molecules. 2022 May 6;27(9):2966. doi: 10.3390/molecules27092966 (PMC9102903; doi:10.3390/molecules27092966)
Supplement: Supplementary file 1 [file molecules-27-02966-s001.zip › Supplementary files/Supplementary file S4 Box plot showing differentially expressed metabolites during the SA stress condition.pdf]

Upregulated intermediate compounds

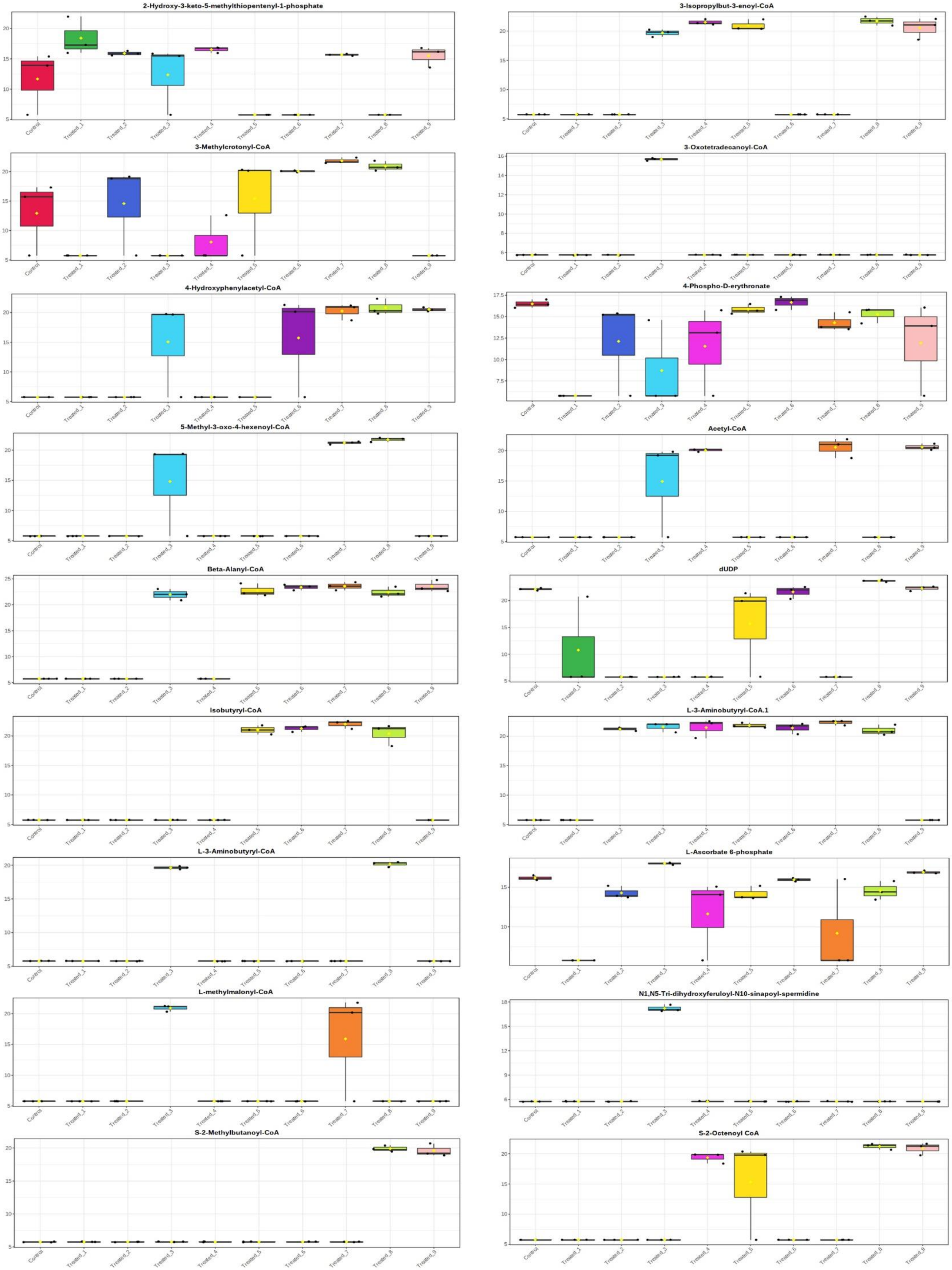

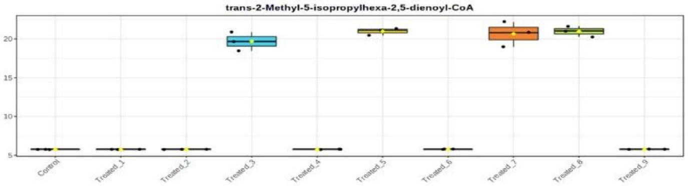

Downregulated intermediate compounds

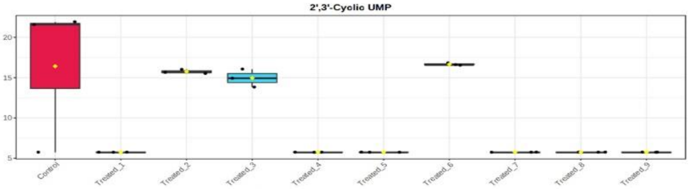

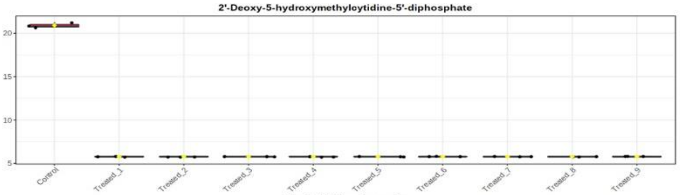

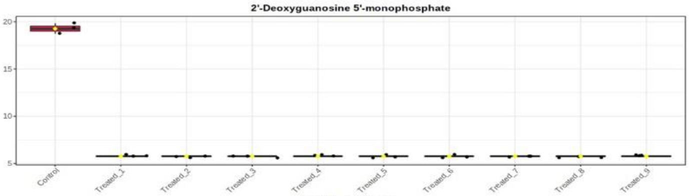

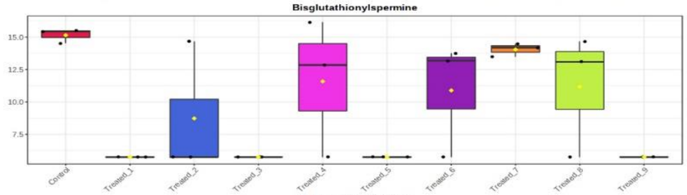

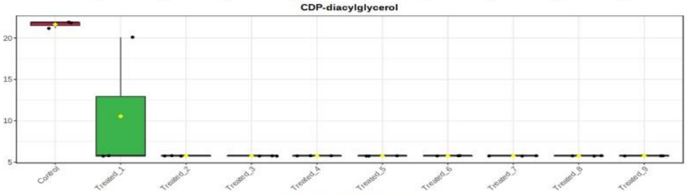

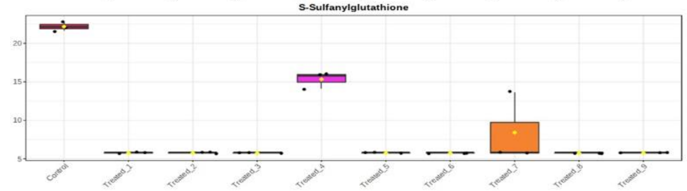

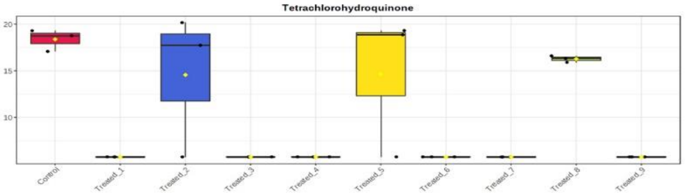

Upregulated Sugars

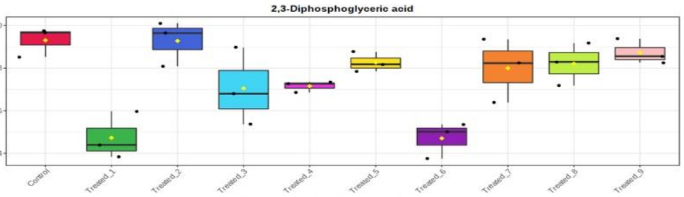

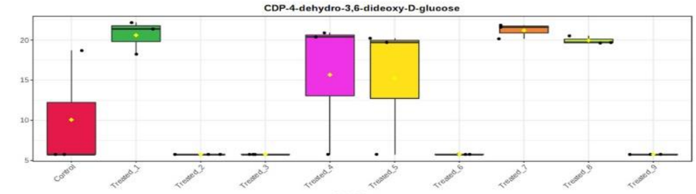

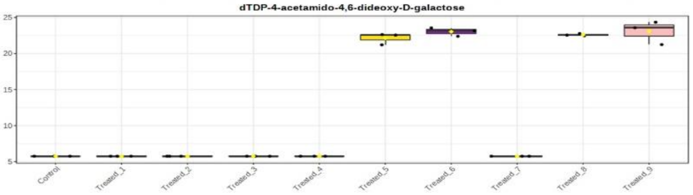

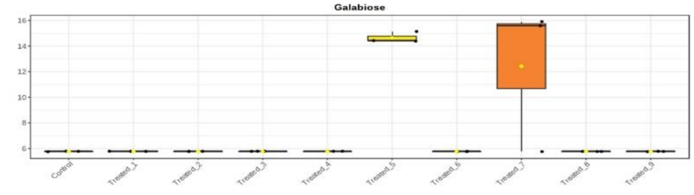

Downregulated Sugars

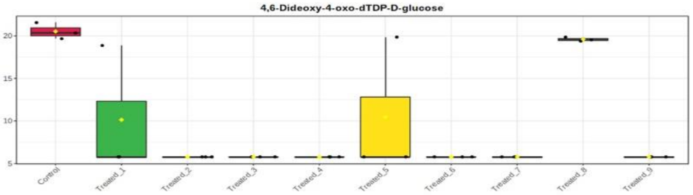

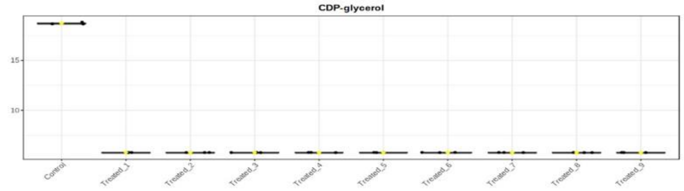

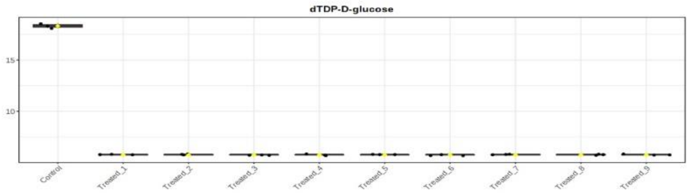

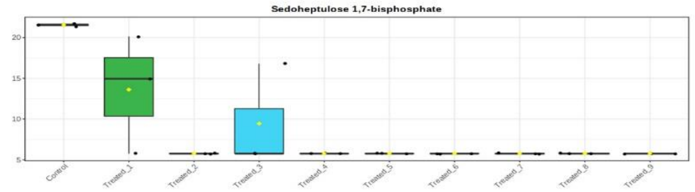

Upregulated Alkaloids

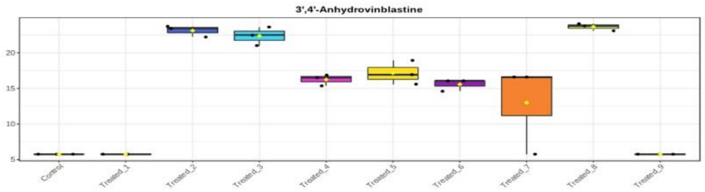

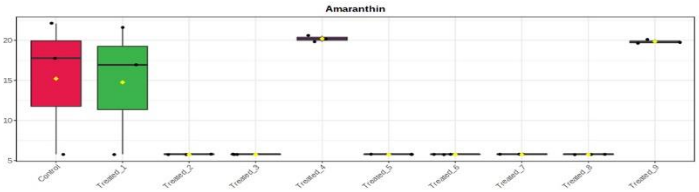

Downregulated Alkaloids

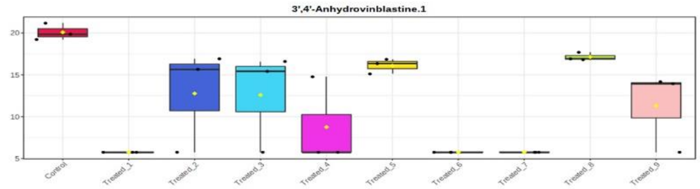

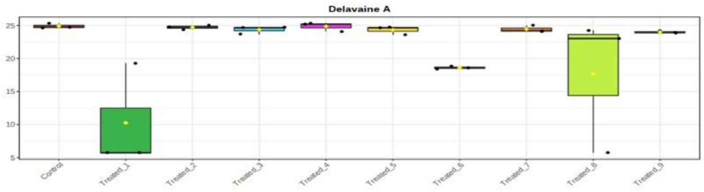

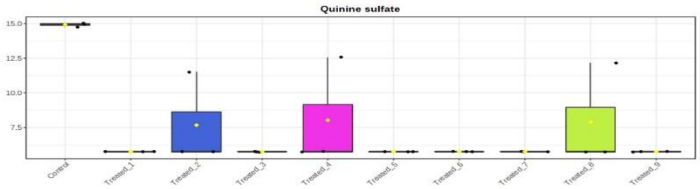

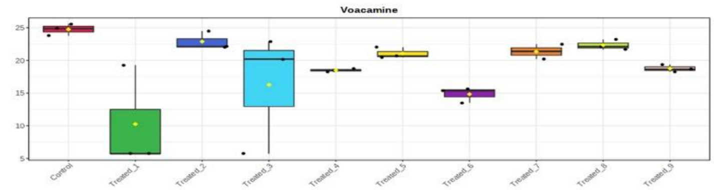

Lignan

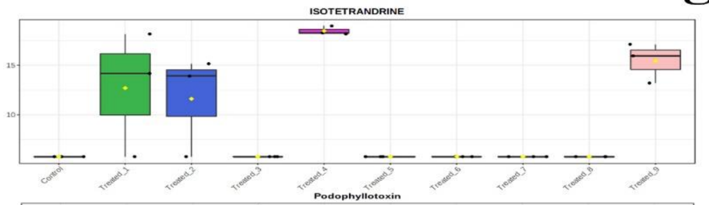

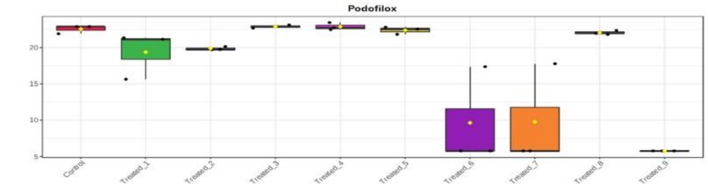

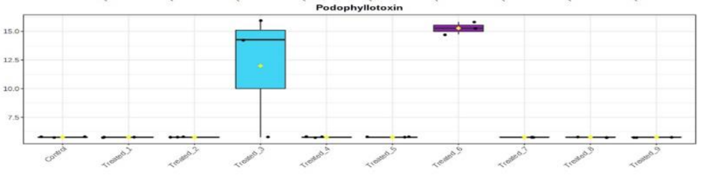

Porphyrin

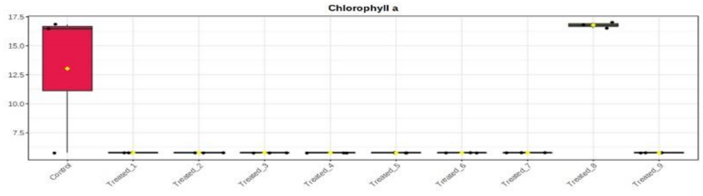

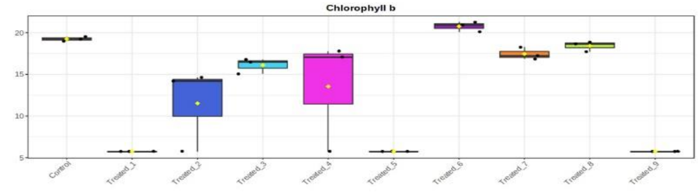

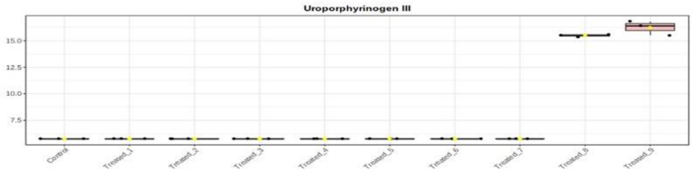

Upregulated Flavanoids

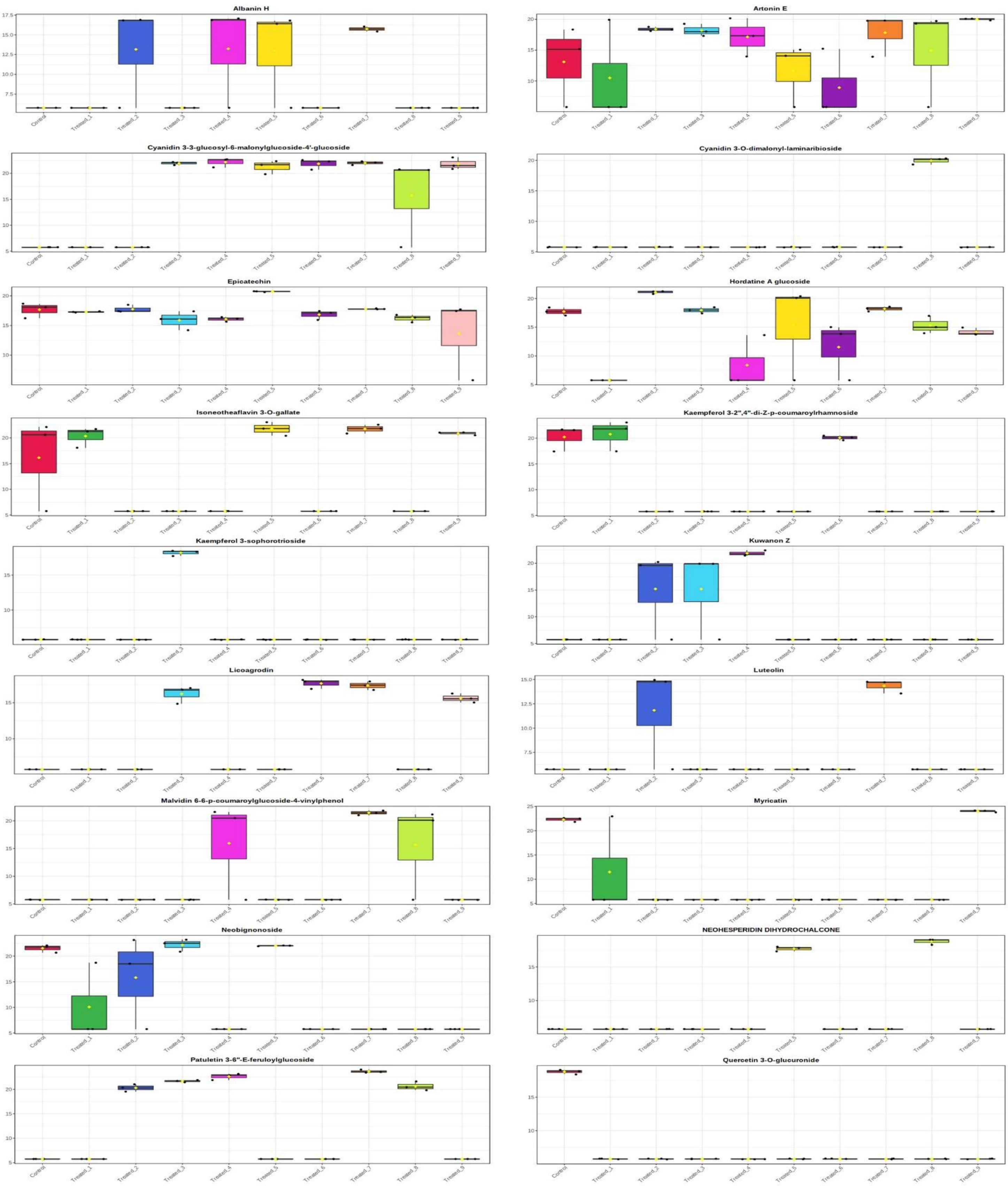

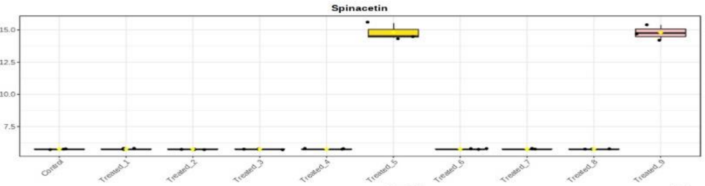

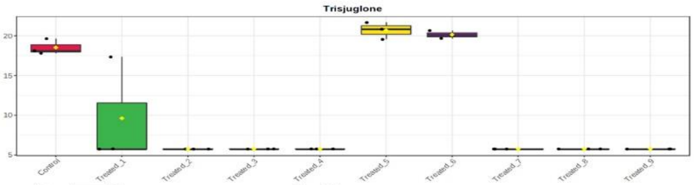

Downregulated Flavanoid

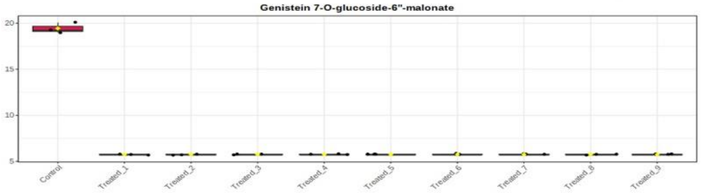

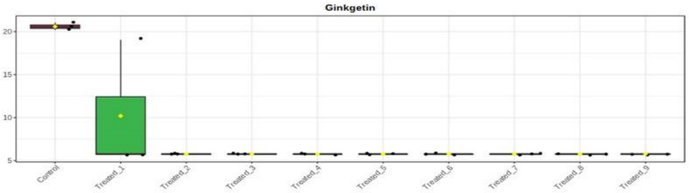

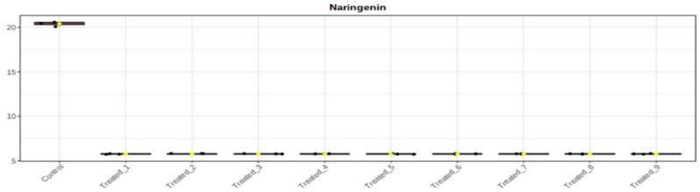

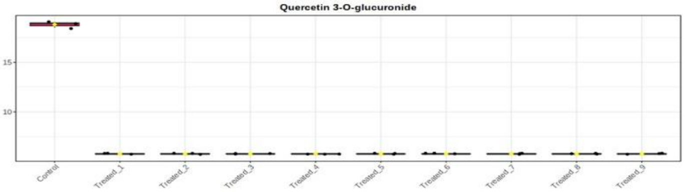

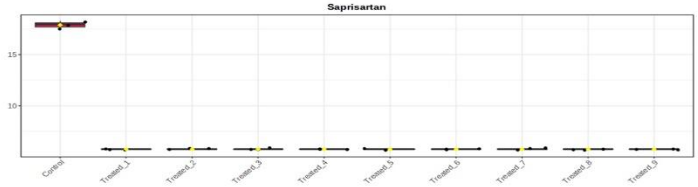

Upregulated Phenol

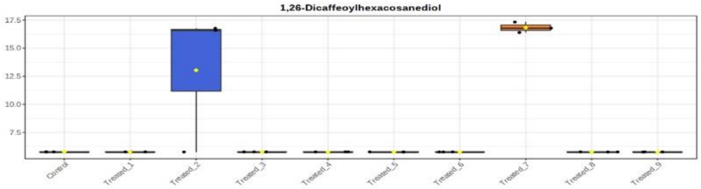

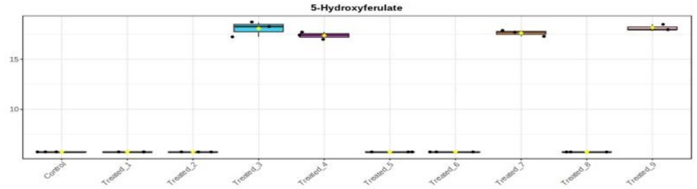

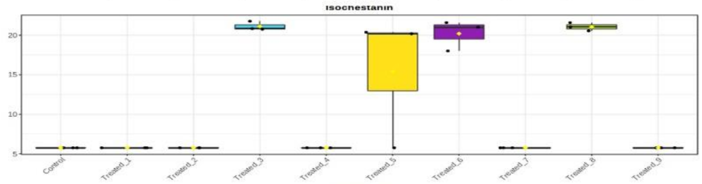

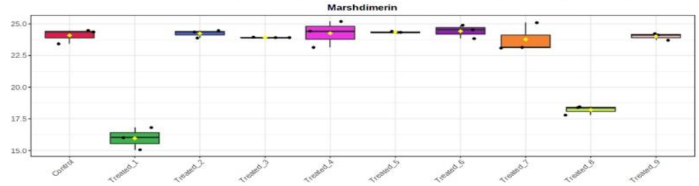

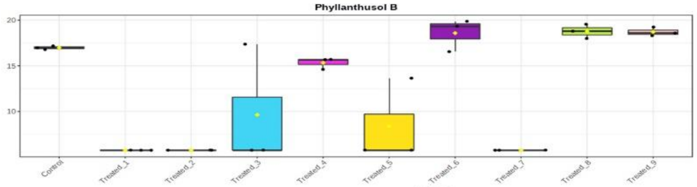

Downregulated Phenol

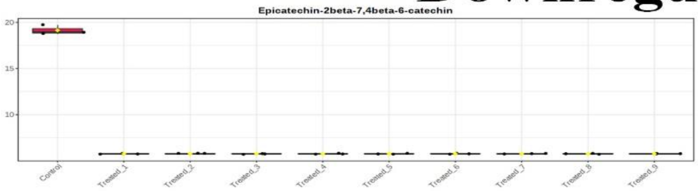

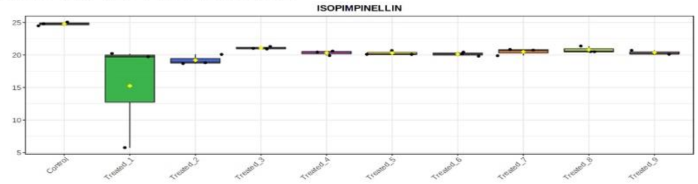

Upregulated Terpenoid

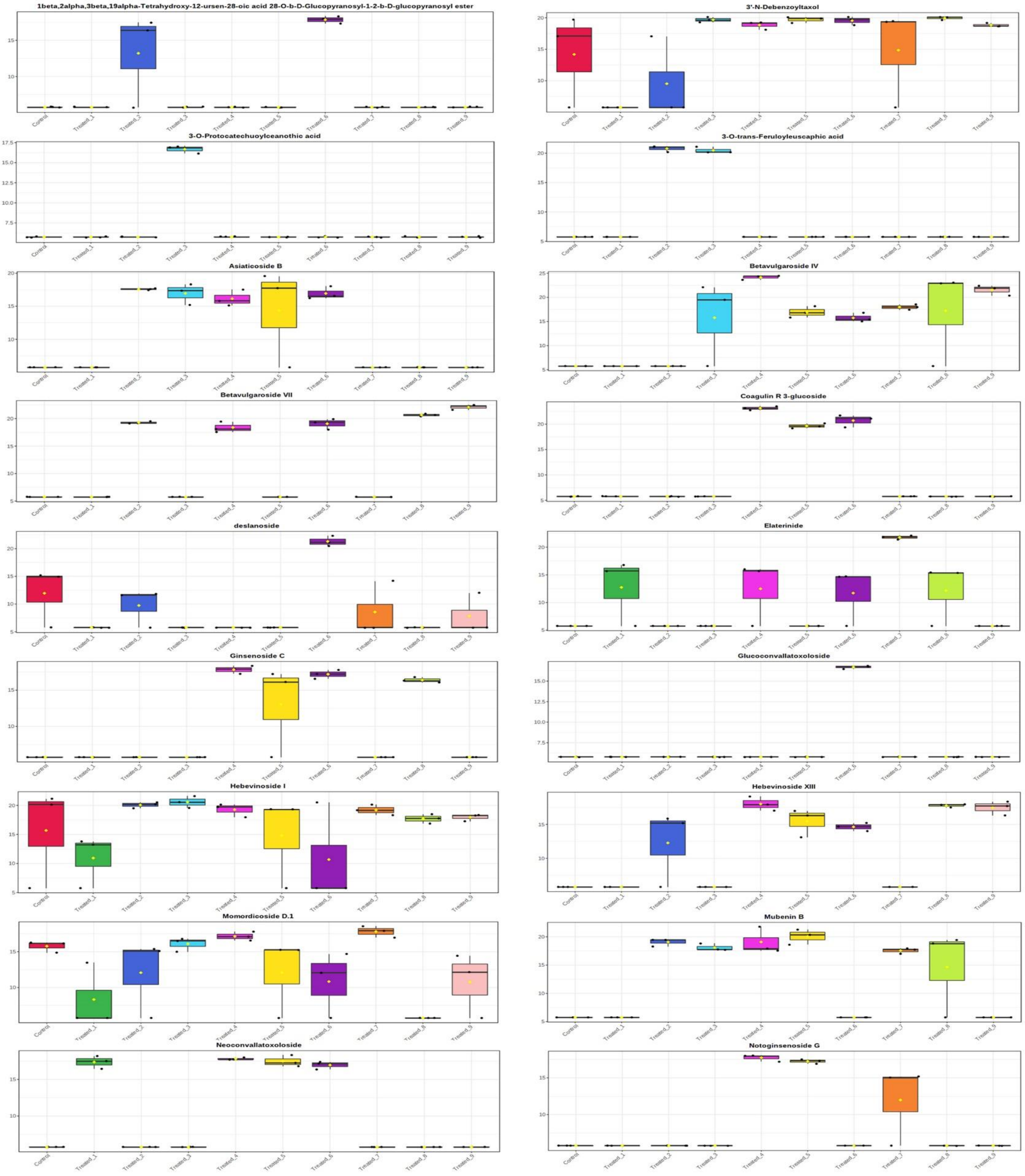

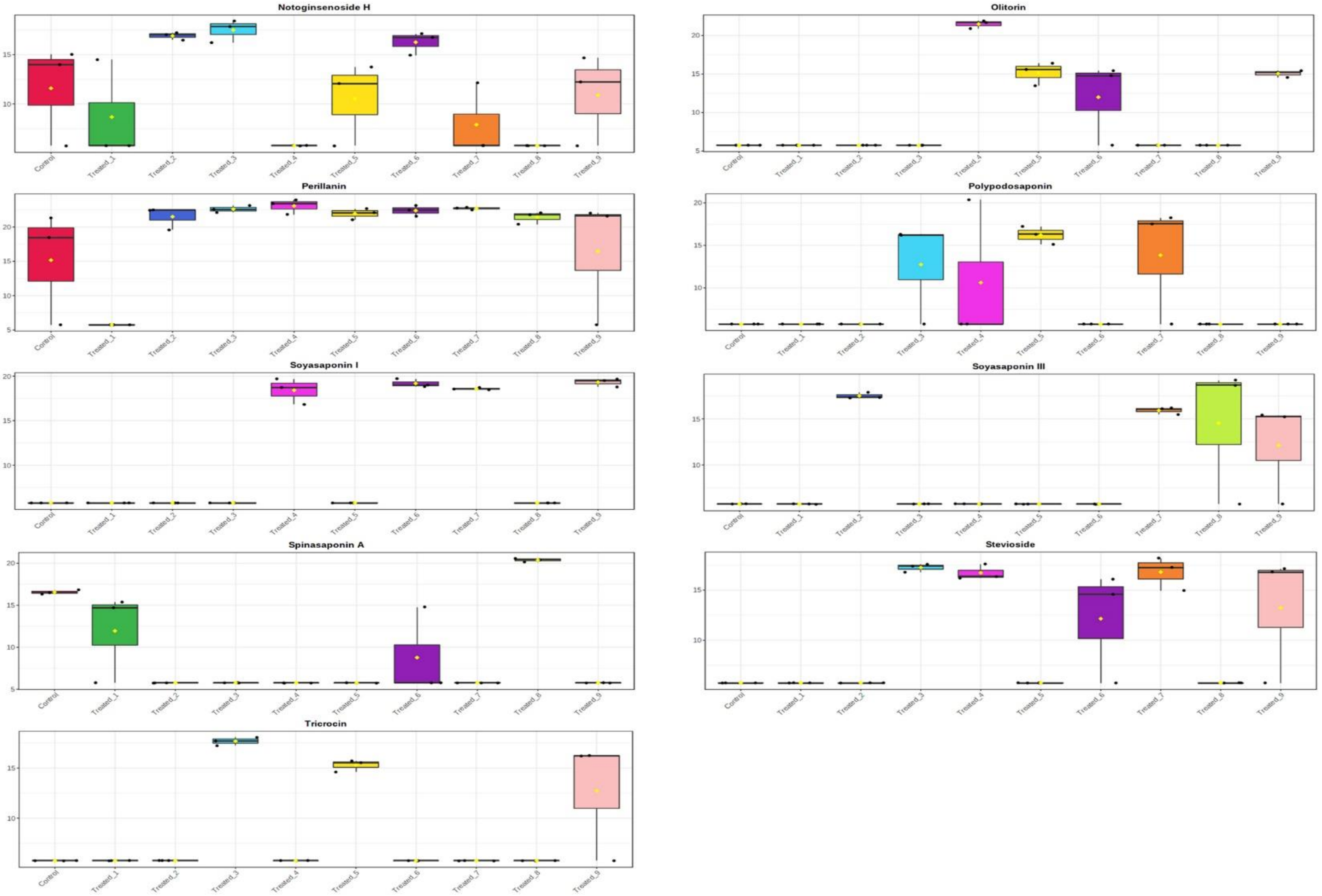

Downregulated Terpenoid

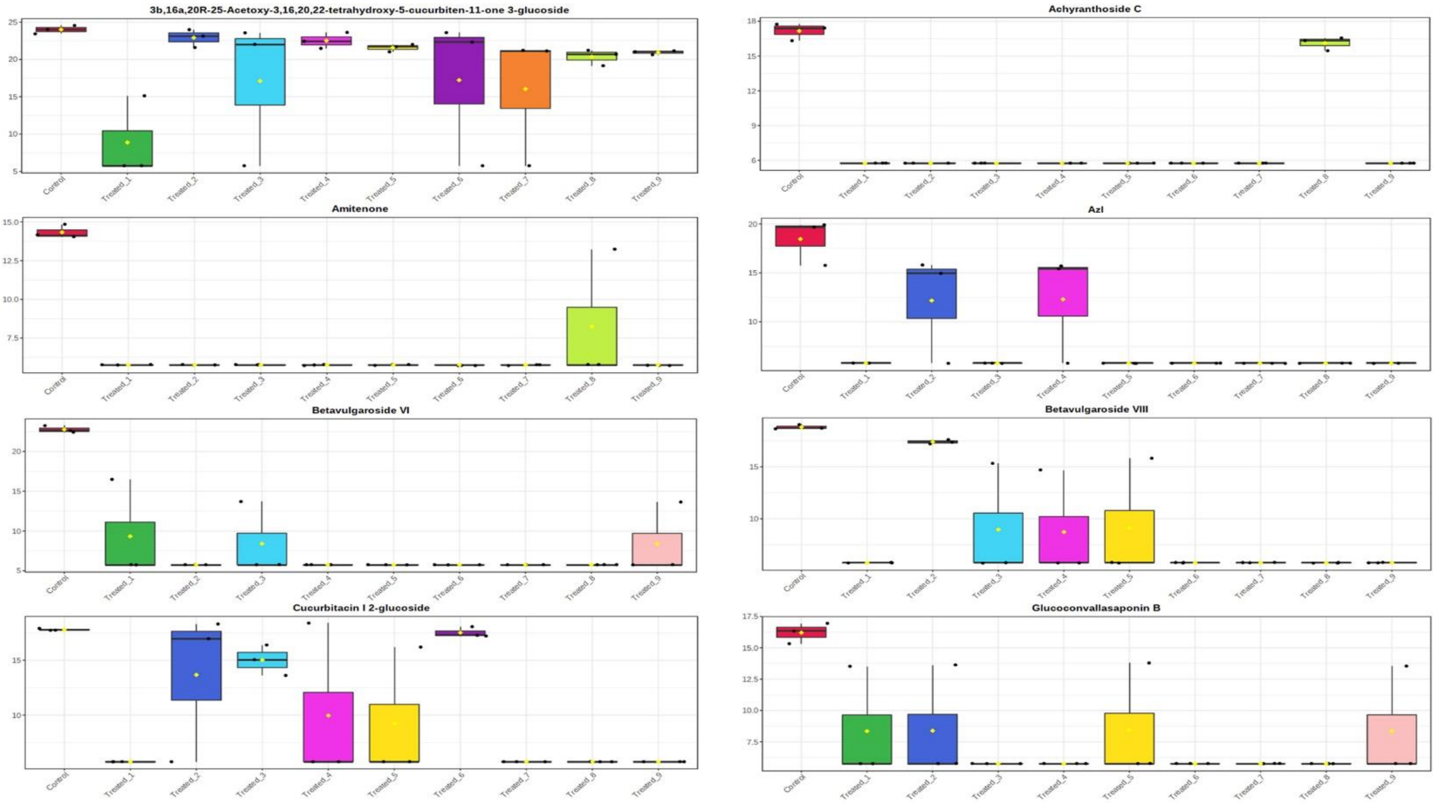

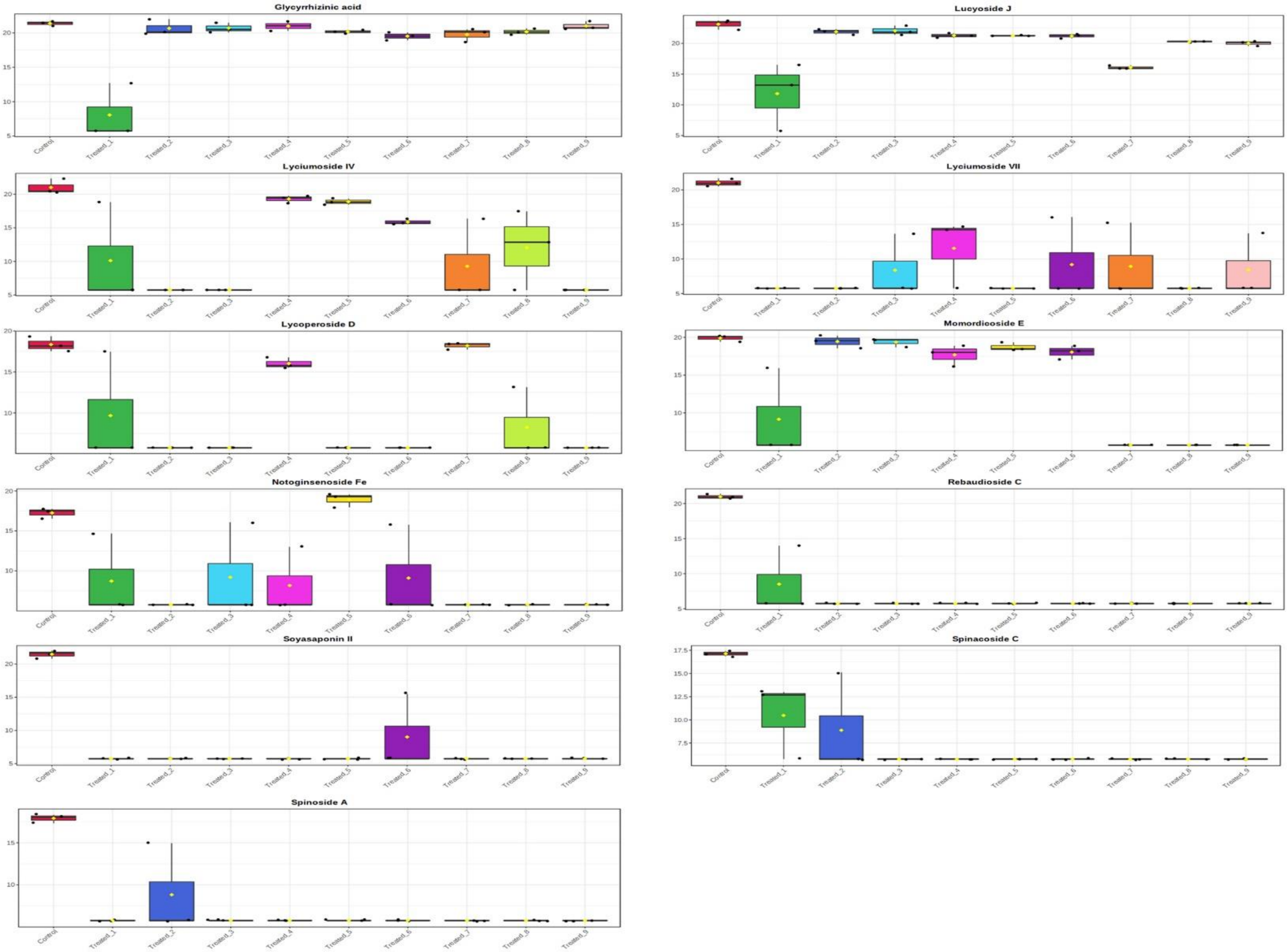
Glycyrrhizinic acid
Lucyoside J
Lyciumoside IV
Lyciumoside VII
Lycoperoside D
Momordicoside E
Notoginsenoside Fe
Rebaudioside C
Soyasaponin II
Spinacoside C
Spinoside A
Control
Treated_1
Treated_2
Treated_3
Treated_4
Treated_5
Treated_6
Treated_7
Treated_8
Treated_9
